# Gendered patterns in the associations between in-school physical activity, classroom peer ecology, and academic achievement among primary school students

**DOI:** 10.1371/journal.pone.0351971

**Published:** 2026-06-22

**Authors:** Anni Byman, Sanna Ulmanen, Kirsi Pyhältö, Heidi Syväoja, Janne Kulmala, Tuija H. Tammelin

**Affiliations:** 1 Faculty of Educational Sciences, University of Helsinki, Helsinki, Finland; 2 Faculty of Education and Culture, Tampere University, Tampere, Finland; 3 Extraordinary Professor, Center for Higher and Adult Education, University of Stellenbosch, Stellenbosch, South Africa; 4 Likes, School of Health and Social Studies, Jamk University of Applied Sciences, Jyväskylä, Finland; Universitatea Transilvania din Brasov, ROMANIA

## Abstract

School is an important setting for increasing students’ engagement in physical activity (PA) and reducing sedentary time (ST), both of which are linked to health and academic achievement. Peer interactions within the classroom can influence students’ engagement in PA and sedentary behaviors during the school day, yet research on how classroom peer ecology relates to objectively measured in-school PA and ST is limited. Even less is known about gender differences in these associations. The objective of this study was to examine how students’ perceived classroom peer ecology is associated with in-school PA and ST and how these factors relate to academic achievement. Gendered patterns in these dynamics were also explored. A sample of 517 Finnish primary school students (53% girls, 47% boys, M^age^ = 11.6 years, SD^age^ = 0.9 years) participated in this cross-sectional study in 2013. Students’ perceptions of classroom peer ecology were investigated through a survey. Moderate-to-vigorous PA (MVPA) and ST during school hours were measured by hip-worn accelerometers. Grade point averages (GPA) were calculated based on subject grades. Associations were investigated through multigroup structural equation modeling analyses. In girls, positive classroom peer relations were associated with lower in-school MVPA, while greater in-school ST was linked to higher GPA. Among boys, higher in-school MVPA was related to higher GPA. Although the MVPA-related associations differed in statistical significance across groups, subsequent formal tests did not indicate statistically significant gender differences, and hence further studies are needed on the subgroup-specific patterns to confirm or invalidate the findings. Efforts to promote a physically active school day could benefit from considering how activity opportunities are experienced within peer dynamics and in relation to gender, including the need for socially inclusive approaches.

## Introduction

Children and adolescents spend a significant portion of their day within the school environment. From a health promotion perspective, school provides an important setting for increasing physical activity (PA) and reducing sedentary time (ST). The health benefits of PA for children and adolescents are well-established [1,2], including beneficial effects on cardiovascular and musculoskeletal health, adiposity, and several components of mental health [2, 3]. The evidence also suggests that PA may positively influence academic achievement [4]. For instance, in-school PA has been associated with performance improvements in mathematics [5] as well as gains in language skills [6]. Yet, most school-aged children do not meet the recommended levels of PA [7], and a substantial part of the school day is spent in sedentary behaviors [8]. Accordingly, it is no surprise that educational practitioners, policymakers, and researchers across the world are interested in finding effective ways to promote students’ PA in school. In Finland, the recent basic education reform endorses the inclusion of a physically active lifestyle as a formal goal of basic education and highlights the schools as key environments for promoting PA and reducing ST [9]. In practice, this can mean increasing opportunities for PA through recess and club activities, as well as incorporating physically active breaks and learning methods into academic lessons.

In schools, the means of promoting PA are primarily socio-pedagogical. Interactions within the school, particularly those with teachers and peers, shape students’ engagement in PA and sedentary behaviors during school hours [10]. PA can be integrated into various segments of the school day, including lessons, recess, lunch breaks, and physical education classes [11]. There is evidence that in-school PA interventions can enhance students’ social behavior and social competence [12, 13]. In general, higher peer support has been linked to higher PA among students in leisure-time settings [14, 15], while higher rates of bullying victimization have been associated with lower PA levels and higher ST levels [16]. Yet, both PA and ST are context-dependent, meaning that the psychosocial correlates of PA and ST may differ between in-school and out-of-school settings [17]. Research on peer support and objectively measured in-school PA remains limited, with findings ranging from positive [18] to non-significant [17,19]. Research on in-school ST and its social correlates has largely been left unexplored, with a recent systematic review [20] identifying only two studies examining ST and social factors, which reported either negative or null associations [21, 22]. Accordingly, not all means of implementing in-school PA automatically enhance positive peer ecology in the classroom. Depending on the classroom dynamics, it may also result in peer-related problems, potentially reducing students’ willingness to engage in physical activities in school [10,23].

Both classroom peer interactions and PA or ST have independently been associated with academic achievement. For example, Tepordei et al. [24] and Yu et al. [25] found that positive peer relationships in the classroom had beneficial effects on academic achievement. These results align with a meta-analytic study by Wentzel et al. [26], which also reported similar associations. Findings regarding in-school PA have been mixed. A systematic review by Rasberry et al. [27] concluded that in-school PA is either positively related to academic achievement or shows no significant relationship, suggesting that adding PA to the school day is unlikely to hinder academic achievement, and may even support it. Similarly, evidence on in-school ST is inconclusive, with a review by Kuzik et al. [20] reporting mixed associations between sedentary behaviors and academic achievement.

Gender differences in in-school PA levels are well-documented: girls tend to be less physically active and more sedentary compared to boys [11,28]. It has also been suggested that associations between social factors and PA differ by gender [29–31]. For example, Sebire et al. [30] found that objectively measured daily PA was negatively associated with peer problems among boys, whereas no such relationship was observed for girls. Milošević and Tubić [29] reported a positive association between perceived social support from friends and higher-intensity overall daily PA among boys only. Marks et al. [31] showed that self-reported PA during school-day breaks was positively associated with having a greater proportion of friends who played sports among girls and a greater proportion of male friends among boys. Overall, these prior findings highlight the need to consider gender differences in research on the social correlates of PA, which could help guide more tailored strategies in future studies or interventions.

**Fig 1:**
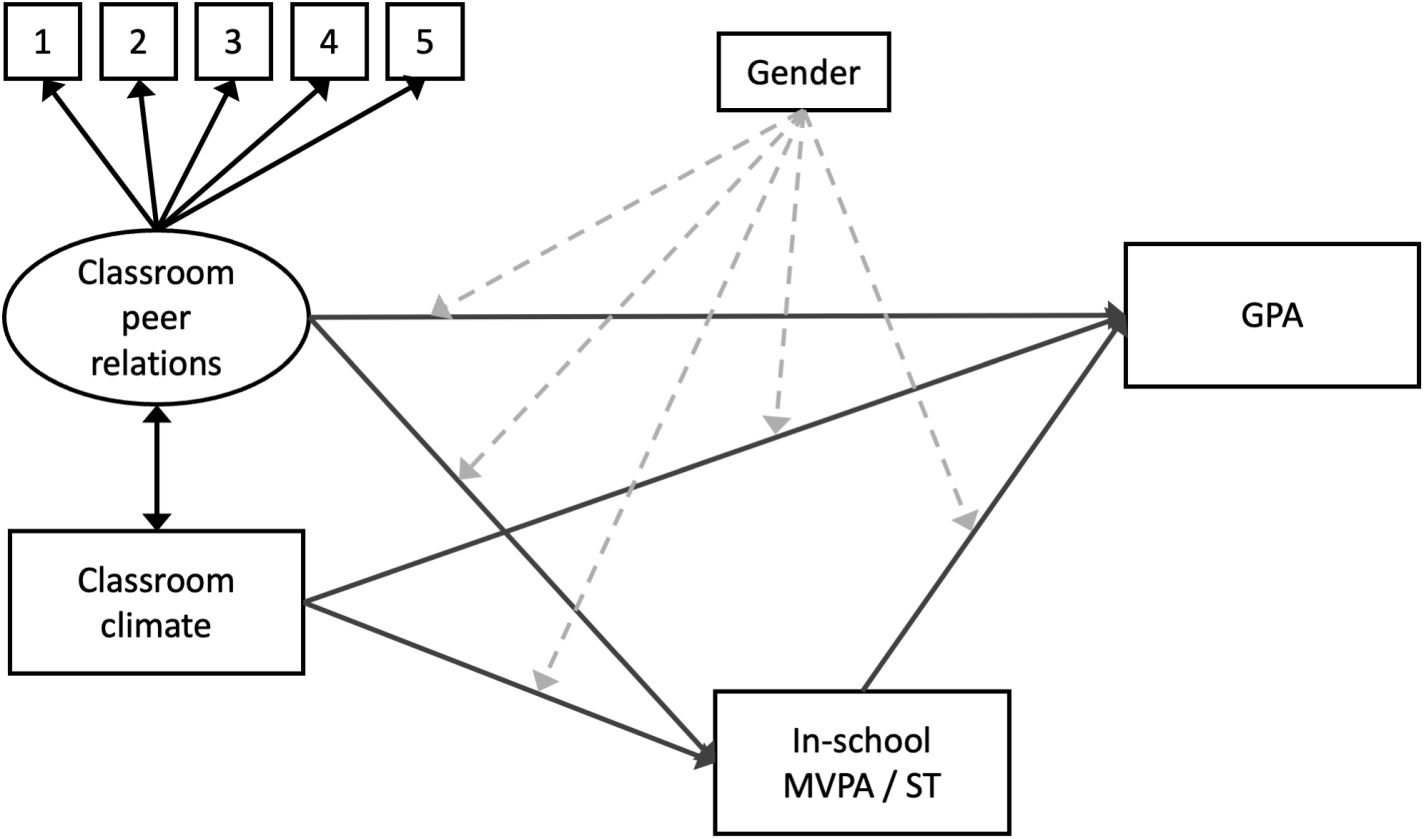
Hypothetical model of the research questions. Note: Two separate models were estimated: one with in-school MVPA and another with in-school ST. A multigroup approach was applied to test whether the associations differed between girls and boys. ST = sedentary time; MVPA = moderate-to-vigorous physical activity; GPA = grade point average.

Despite a growing body of research on student peer outcomes and PA, much of it focuses on overall PA [29,32] or PA-specific peer support, which captures more narrowly defined behaviors directly related to PA (e.g., encouragement, shared activities, team sports participation) [17–19,33]. In contrast, research on how broader classroom social contexts is associated with in-school PA and ST is still in its infancy [3,34], and even less is known about potential gender differences in these relationships. The current study focuses on students’ individual perceptions of the classroom peer ecology as a broader social context, including peer relations in the classroom and classroom climate. Classroom climate reflects overall perceptions of the classroom environment, whereas peer relations refer more specifically to the quality of relationships among classmates, including acceptance, friendliness, and support. Accordingly, we examined how the perceived classroom peer ecology relates to students’ in-school PA and ST and academic achievement. The study offers a novel perspective by examining these dynamics and potential gender differences in the interrelations among Finnish primary school students. The hypothetical model is illustrated in Fig 1, and the following research questions were addressed:

RQ1) How are perceived classroom peer relations and classroom climate related to in-school moderate-to-vigorous physical activity (MVPA), in-school ST, and academic achievement among primary school students?

RQ2) Do students’ in-school MVPA or ST mediate the relationship between (a) classroom peer relations and academic achievement and (b) classroom climate and academic achievement?

RQ3) Are there gendered differences in these interrelations?

## Materials and Methods

### Primary education in Finland

In Finland, primary education is part of comprehensive schooling, covering grades 1–9 for children aged 7–16. Primary education includes grades 1–6 (usually ages 7–13), and most subjects are taught by the same class teacher. Education is free and publicly funded, including textbooks and daily school meals. All schools followed the 2004 edition of the National Core Curriculum for Basic Education [35], with schools and teachers having considerable autonomy in its implementation. Students in grades 4–6 received approximately 1.5 hours of curriculum-mandated PA per week through compulsory physical education (2 x 45-minute lessons).

### Participants and procedures

Data for this cross-sectional study were collected in 2013 as part of a larger research project called Students’ Physical Activity and Well-being [36–38]. Participants were recruited from schools selected across various regions, representing both large urban cities and smaller towns. The recruitment period for this study was from January 1, 2013, to May 31, 2013. In total, 1710 children in grades 4–7 were invited to participate in the larger research project, with 970 (56.7%) agreeing to do so. Of these, 880 participants (90.7%) had valid accelerometer data, corresponding to 51.4% of the total invited sample. The current study focused on primary school students with valid accelerometer data. The sample comprised 517 students in grades 4–6 (276 girls, 241 boys, M^age^ = 11.6 years, SD^age^ = 0.9 years) from six schools and 42 classrooms across Finland.

During a regular school day, students filled out a questionnaire with measures of study variables and demographic information. Students were given accelerometers along with instructions on how to use them. Ethics approval for this study was obtained from the Ethics Committee of the University of Jyväskylä (11.12.2012/ T.H.T.). It followed the principles of the Declaration of Helsinki. Participation in the study was voluntary, and all participants and their guardians provided written informed consent prior to participation.

### Measures and variables

#### Classroom peer relations.

Classroom peer relations were assessed with five items capturing students’ perceptions of the quality of peer interactions, including prosocial behavior, sense of belonging, and mutual respect. (Table 1) [39]. Items were rated with a five-point Likert scale ranging from 1 (strongly disagree) to 5 (strongly agree). The scale showed good internal consistency (α = .81). Structural validity was supported by confirmatory factor analysis (Table 2). Test–retest reliability was assessed among 181 students in grades 4–9 using a two-week interval as described earlier [40]. The scale showed good repeatability (ICC = 0.81, 95% CI: 0.76–0.86), with single-item ICCs ranging from 0.66 to 0.71.

**Table 1:** Classroom peer relations: First-order factor and corresponding items.

First-order factor	Questionnaire items
Classroom peer relations	1) The students in my class get along well together 2) Most of the students in my class are friendly and helpful 3) Other students accept me as I am 4) When a student in my class is sad, some of the classmates try to help 5) The students in my class are respectful towards others

**Table 2:** Model fit for classroom peer relations across gender.

Model	χ² (df)	CFI	TLI	RMSEA (90% CI)	SRMR	Δχ^2^(df)	ΔCFI	ΔRMSEA
Girls	9.58 (5)	.99	.98	.06 (0.00–0.11)	.02			
Boys	13.14 (5)*	.96	.92	.08 (0.03–0.13)	.04			
Configural	20.64 (9)*	.98	.96	.07 (0.03–0.11)	.03	–	–	–
Metric	27.71 (14)*	.98	.97	.06 (0.03–0.09)	.06	6.10 (5)	−0.00	−0.01
Partial scalar	38.68 (17)**	.97	.96	.07 (0.04–0.10)	.08	12.30 (3)**	−0.01	0.01
Scalar	80.35 (19)***	.90	.90	.11 (0.09–0.14)	.10	62.96 (14)***	−0.08	0.05

Note: χ2 = chi-squared test of exact fit; df = degrees of freedom; CFI = comparative fit index; TLI = Tucker–Lewis index; RMSEA = root mean square error of approximation; 90% CI = 90% confidence interval of the RMSEA; SRMR = standardized root mean squared residual; Δχ^2^ = Satorra-Bentler scaled chi-square difference test, *p < .05, **p < .01, ***p < .001.

#### Classroom climate.

Classroom climate was measured with a question modified from the home climate question in HBSC surveys [39,41] by replacing the word ‘home’ with ‘class’. Students were asked, “How do you experience the climate in your class?”. Students responded to the questions on a five-point scale ranging from 1 (very poor) to 5 (very good). The item was included to capture students’ general perceptions of the classroom environment and to complement the more detailed measure of classroom peer relations. Brief single-item measures may also reduce participant burden and can provide a straightforward indicator with acceptable face validity in large school-based surveys. Although single-item measures cannot fully capture the multidimensional nature of classroom climate, similar brief indicators have been used in prior research [41]. The item demonstrated good test–retest reliability (ICC = 0.73, 95% CI: 0.66–0.79) and showed a positive correlation with the latent classroom peer relations construct, consistent with theoretical expectations.

#### In-school MVPA and ST.

In-school MVPA and ST were measured with ActiGraph GT3X+ and wGT3X+ accelerometers (ActiGraph, Pensacola, Florida, USA) and described in more detail by Kallio et al. [37]. Accelerometers have been shown to provide valid and reliable estimates of children’s PA and ST [42, 43]. Students were instructed to wear the device on the right side of the hip during waking hours for seven consecutive days and to take it off only during water-based activities. Students completed diaries to report on the school’s start and end times for each measurement day. Data were collected with a sampling frequency of 30 Hz, and the raw accelerometer data were converted into 15-second intervals. The data were exported using ActiLife software (release 5.0 or later). Data reduction was performed by using a Visual Basic Macro in Excel. In-school MVPA and ST were distinguished by using time filters specific to each student based on their diaries. Non-wear time was defined as a period with at least 30 consecutive minutes of zero counts [44, 45]. To determine time spent in MVPA and sedentary behaviors, the following thresholds were applied: < 25 counts per 15 s for ST and ≥574 counts per 15 s for MVPA [46]. In-school MVPA and ST were calculated and expressed as minutes per school hour with respect to daily school time. At least two valid measurement days were required to be included in further analysis. Wearing time had to be at least 80% of school time to be considered valid [47, 48].

#### Academic achievement.

Teacher-rated grades in school subjects, including academic subjects as well as arts and physical education, were received from education services, and grade point averages (GPAs) were calculated as the mean of individual grades to indicate overall academic achievement [49]. In Finnish comprehensive schools, grades range from 4 (failure) to 10 (excellent knowledge and skills).

### Statistical analysis

Descriptive statistics and variable correlations were calculated using IBM SPSS Statistics (version 29.0).

The structural validity of the classroom peer relations measurement model was first assessed using confirmatory factor analysis (CFA), and the hypothetical models were tested with structural equation modeling (SEM). All CFA and SEM analyses were conducted in Mplus (version 8.11) [50] using the MLR estimator, which applies maximum likelihood estimation with standard errors and chi-square statistics robust to non-normality [50]. Missing data ranged from 1.4% to 20.7%, with the sample size (n) for each study variable reported in Table 3. Little’s MCAR test [51] indicated that the data were not missing completely at random, χ² (10) = 24.93, *p* = .005. Therefore, missing values were managed in Mplus by using full information maximum likelihood (FIML), which incorporates all available data and provides unbiased estimates under the assumption of data missing at random.

**Table 3:** Observed variable correlations, means, and standard deviations for the whole sample.

Variables	1	2	3	4	5
1 Classroom peer relations	1				
2 Classroom climate	.52***	1			
3 In-school MVPA	−.07	.04	1		
4 In-school ST	.03	−.05	−.70***	1	
5 GPA	.05	.05	−.02	.15**	1
Mean	3.8	3.9	3.9	38.6	8.3
SD	0.7	0.9	1.7	4.6	0.7
min-max	1–5	1–5	0.4–12.4	22.3–49.8	5.5–9.8
ICC	0.24	0.16	0.30	0.28	0.06
n	509	510	517	517	410

Note: MVPA = moderate-to-vigorous physical activity; ST = sedentary time; GPA = grade point average; SD = standard deviation; ICC = intraclass correlation coefficient; **p < .01, ***p < .001.

Because the data were nested within classrooms, intraclass correlation coefficients (ICCs) were calculated to estimate class-level variance. The ICCs indicated moderate clustering in classroom peer relations, classroom climate, in-school MVPA, and ST, whereas GPA showed minimal clustering. Multigroup cluster-robust SEM models were explored to account for the nesting of students within classrooms. However, given the number of classrooms relative to the complexity of the multigroup SEM, cluster-robust estimation was not reliable for all parameters. Because gender differences were a focus of the study, the primary analyses were conducted using single-level multigroup SEM and are interpreted as individual-level associations. To assess the robustness of the findings, sensitivity analyses were conducted by estimating gender-specific models using cluster-robust standard errors (TYPE = COMPLEX in Mplus). These analyses yielded substantively similar patterns of results, with the key associations remaining statistically significant (S1 Appendix). The evaluation of model fit was based on predefined thresholds. Acceptable model fit was indicated by a comparative fit index (CFI) and Tucker–Lewis index (TLI) greater than 0.90, as well as a root mean square error of approximation (RMSEA) and standardized root mean square residual (SRMR) of 0.08 or less [52, 53].

### CFA

CFAs were first conducted separately for girls and boys, both showing good model fit (Table 2). Measurement invariance across gender was then examined by using multigroup CFAs in a stepwise approach: configural, metric, and scalar models [54]. Invariance was evaluated using ΔCFI ≤ 0.01 and ΔRMSEA ≤ 0.015, with chi-square difference tests reported but interpreted cautiously due to sensitivity to sample size [55, 56]. The configural model showed good fit, indicating the same factor structure across gender. Next, metric invariance was tested by constraining factor loadings to be equal across gender. Metric invariance was also supported, suggesting that the constructs were similarly understood across gender. However, the scalar model showed poor fit, with a significant chi-square difference test and changes in CFI and RMSEA exceeding recommended cutoffs, indicating scalar non-invariance. To address this, a partial scalar model was tested by freeing intercepts for items 1 and 4, based on Mplus model modification indices. This model showed acceptable model fit, and while the changes in CFI and RMSEA were within acceptable thresholds, the significant chi-square difference test provided only limited support for partial scalar invariance. As metric invariance was established in CFA and this level of invariance was adequate for the study’s aims, it was used in subsequent SEM analyses. All CFA results are shown in Table 2.

### SEM

Two separate models were estimated: one with in-school MVPA and another with in-school ST (Fig 1). They were modeled separately due to their conceptual distinctions and their strong inverse correlation, which could complicate interpretation if included in the same model. Age was included as a covariate in both models, with regression paths specified from age to in-school MVPA and in-school ST, given the well-established associations between age and children’s PA and sedentary behavior [57]. A multigroup approach was applied to test whether the associations differed between girls and boys. In this approach, the same structural model was estimated simultaneously for both groups, allowing for a formal comparison of path coefficients to determine potential gender differences in the strength or direction of the relationships among variables. To test whether the gender differences were statistically significant, significant regression paths were individually constrained to be equal across groups. Model comparisons were then conducted using the Satorra–Bentler scaled chi-square difference test, which assesses whether equality constraints significantly worsen model fit.

## Results

Descriptive statistics of the study variables are presented in Tables 3 and 4. Across the full sample, the strongest correlation was a negative one between in-school MVPA and in-school ST. Classroom climate showed a positive correlation with peer relations. In addition, in-school ST was positively correlated with GPA (Table 3). Overall, classroom peer relations and classroom climate were perceived as good by girls and boys, and no significant gender differences were found in the social variables (Table 4). Boys spent more time in MVPA and less time in ST during school hours, whereas girls had higher GPAs.

**Table 4:** Gender-specific descriptive statistics and gender differences in study variables.

	Girls (n = 276)				Boys (n = 241)					
	n	M	SD	min–max	n	M	SD	min–max	Cohen’s d^a^	*p* ^b^
**Social factors**										
Classroom peer relations^c^	272	3.8	0.7	1–5	237	3.7	0.7	1–5	0.14	.06
Classroom climate	272	4.0	0.8	1–5	238	3.9	0.9	1–5	0.12	.70
**In-school PA + ST**										
Wear time per day (min)	276	305.8	33.1	169.9–420.0	241	303.7	31.3	180.0–370.0	0.07	.47
MVPA (min/h)	276	3.1	1.4	0.4–9.3	241	4.7	1.7	1.3–12.4	−1.03	<.001***
ST (min/h)	276	40.5	4.2	28.9–49.8	241	36.4	4.0	22.3–47.0	1.00	<.001***
**Academic achievement**										
GPA	216	8.5	0.6	6.9–9.8	194	8.2	0.7	5.5–9.5	0.46	<.001***

Note: ^a^ positive values indicate higher scores among girls; ^b^ *p*-value for gender differences (independent samples t-test); ^c^ average score of the five items; *** *p* < .001; PA = physical activity; ST = sedentary time; MVPA = moderate-to-vigorous physical activity; GPA = grade point average; M = mean; SD = standard deviation.

### In-school MVPA and its relations to classroom peer relations, classroom climate, and academic achievement

**Fig 2:**
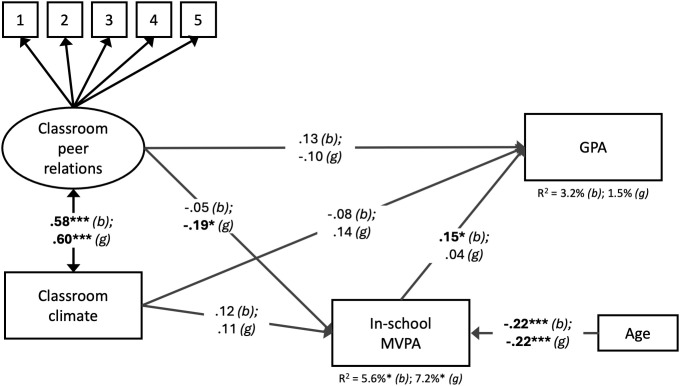
SEM results regarding in-school MVPA. Note: MVPA = moderate-to-vigorous physical activity; GPA = grade point average; *p < .05, ***p < .001; b = boys, g = girls.

The hypothesized model demonstrated good fit to the data (χ^2^ (52) = 85.91, *p* = .002; CFI = 0.96; TLI = 0.95; RMSEA = 0.05, 90% CI [0.03–0.07]; SRMR = 0.06). Among girls, classroom peer relations were directly and negatively associated with in-school MVPA (−0.19). Among boys, in-school MVPA was positively associated with GPA (0.15). No other paths between the study variables reached statistical significance, nor were any significant mediating effects observed. Results are presented in Table 5 and Fig 2. A model comparison showed that constraining the significant paths to be equal across genders did not significantly worsen model fits (both *p* > .05). Thus, although parameter estimates differed between boys and girls, the results suggest that the associations did not significantly differ by gender.

**Table 5:** Standardized estimates, confidence intervals, and *p*-values of hypothesized model (in-school MVPA).

	Estimate	95% CI	SE	*p*
Effects of peer relations on in-school MVPA				
Girls	−0.19	−0.36; −0.03	0.08	.023*
Boys	−0.07	−0.25; 0.10	0.09	.411
Effects of peer relations on GPA				
Girls	−0.10	−0.33; 0.13	0.12	.382
Indirect (through MVPA)	−0.01	−0.03; 0.02	0.01	.553
Total	−0.11	−0.33; 0.11	0.11	.330
Boys	0.14	−0.06; 0.33	0.10	.177
Indirect (through MVPA)	−0.01	−0.04; 0.02	0.02	.475
Total	0.12	−0.07; 0.32	0.10	.215
Effects of classroom climate on in-school MVPA				
Girls	0.09	−0.06; 0.23	0.08	.254
Boys	0.11	−0.04; 0.26	0.08	.159
Effects of classroom climate on GPA				
Girls	0.14	−0.08; 0.35	0.11	.218
Indirect (through MVPA)	0.00	−0.01; 0.02	0.01	.578
Total	0.14	−0.07; 0.35	0.11	.199
Boys	−0.08	−0.25; 0.09	0.09	.362
Indirect (through MVPA)	0.02	−0.01; 0.04	0.01	.277
Total	−0.06	−0.23; 0.10	0.08	.458
Effects of in-school MVPA on GPA				
Girls	0.04	−0.09; 0.18	0.07	.544
Boys	0.15	0.02; 0.28	0.07	.027*
Effects of age on in-school MVPA				
Girls	−0.22	−0.34; −0.10	0.06	<.001***
Boys	−0.22	−0.33; −0.11	0.06	<.001***

Note: MVPA = moderate-to-vigorous physical activity; GPA = grade point average; SE = standard error; *p < .05, ***p < .001.

The explained overall variance was modest across outcomes based on R² estimates. The model accounted for 5.6% of the variance in in-school MVPA among boys (p = .031) and 7.2% among girls (p = .023), whereas the variance explained in GPA was 3.2% (p = .238) and 1.5% (p = .462), respectively.

### In-school ST and its relations to classroom peer relations, classroom climate, and academic achievement

**Fig 3:**
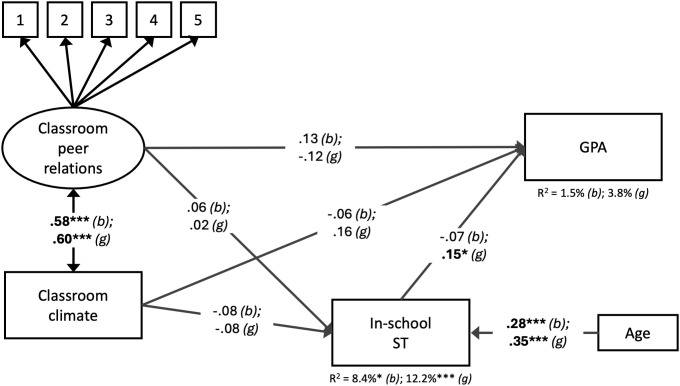
SEM results regarding in-school ST. Note: ST = sedentary time; GPA = grade point average; *p < .05, ***p < .001; b = boys, g = girls.

The model fit the data well (χ^2^ (52) = 85.22, *p* = .003; CFI = 0.96; TLI = 0.95; RMSEA = 0.05, 90% CI [0.03–0.07]; SRMR = 0.06). Standardized regression estimates showed that in-school ST was positively associated with GPA among girls (0.15), whereas the association was negative and non-significant among boys (−0.07). A model comparison showed that constraining the significant path to be equal across genders worsened model fit, Δχ² (1) = 6.31, *p* = .012. No additional associations between the study variables were statistically significant, and no significant mediation effects were detected. SEM results are presented in Table 6 and Fig 3.

**Table 6:** Standardized estimates, confidence intervals, and p-values of hypothesized model (in-school ST).

	Estimate	95% CI	SE	*p*
Effects of peer relations on in-school ST				
Girls	0.03	−0.14; 0.20	0.09	.750
Boys	0.08	−0.09; 0.26	0.09	.338
Effects of peer relations on GPA				
Girls	−0.12	−0.33; 0.09	0.11	.269
Indirect (through ST)	0.00	−0.02; 0.03	0.01	.744
Total	−0.12	−0.33; 0.10	0.11	.300
Boys	0.13	−0.07; 0.33	0.10	.191
Indirect (through ST)	−0.01	−0.02; 0.01	0.01	.528
Total	0.13	−0.07; 0.32	0.10	.208
Effects of classroom climate on in-school ST				
Girls	−0.04	−0.22; 0.15	0.10	.685
Boys	−0.07	−0.22; 0.08	0.08	.346
Effects of classroom climate on GPA				
Girls	0.16	−0.04; 0.35	0.10	.125
Indirect (through ST)	−0.01	−0.03; 0.02	0.01	.678
Total	0.15	−0.06; 0.36	0.11	.159
Boys	−0.06	−0.23; 0.10	0.09	.454
Indirect (through ST)	0.01	−0.01; 0.02	0.01	.528
Total	−0.06	−0.22; 0.11	0.08	.483
Effects of in-school ST on GPA				
Girls	0.15	0.02; 0.28	0.07	.020*
Boys	−0.07	−0.20; 0.07	0.07	.332
Effects of age on in-school ST				
Girls	0.35	0.25; 0.45	0.05	<.001***
Boys	0.28	0.17; 0.39	0.05	<.001***

Note: ST = sedentary time; GPA = grade point average; SE = standard error; *p < .05, ***p < .001.

As in the model with in-school MVPA, the explained variance was modest across outcomes. The model accounted for 8.4% of the variance in in-school ST among boys (p = .010) and 12.2% among girls (p < .001), whereas the variance explained in GPA was 1.5% (p = .456) for boys and 3.8% (p = .223) for girls.

## Discussion

The objective of this study was to examine how perceived classroom peer ecology is associated with in-school PA and ST and how these factors relate to academic achievement among primary school students. The findings identified some gendered patterns in the dynamics between the perceived peer ecology, in-school MVPA, in-school ST, and academic achievement.

In the current study, more positive perceptions of classroom peer relations were significantly associated with less time spent in in-school MVPA among girls, whereas this association was not statistically significant among boys. Although the association observed differed in statistical significance across groups, subsequent tests of gender differences were not statistically significant, and thus the findings concerning gendered pattern should be interpreted cautiously. Previous research shows that social dynamics can shape students’ engagement in in-school PA [10], and the relationship between social dynamics and activity behaviors may vary by gender [29–31]. While prior studies have suggested that PA can support social competence and peer relationships [12, 13] and that peer-related social factors are positively associated with higher PA levels [14,15,41], the present study did not find evidence linking more positive classroom peer dynamics with in-school MVPA.

This unexpected finding may suggest a potential inverse association between self-reported peer relations in the classroom and engagement in more intense forms of in-school PA, particularly in the descriptive pattern observed among girls. One possible explanation for the finding is that girls may shy away from higher-intensity physical activities at school to avoid potential social discomfort or exclusion. The interpretation, though speculative, is aligned with earlier research suggesting that peer-related challenges can be more pronounced during PA and may act as barriers to participation [10,23]. Prior research shows that boys’ daily physical activities are often more vigorous, whereas girls’ activities tend to be less physically intense and more socially oriented [58]. Stearns et al. [59] also suggest that among girls, school friends tend to have similar PA levels, rather than friendship consistently leading to higher PA levels. Thus, similarities in PA patterns may attract and sustain friendships. Accordingly, stronger peer relations among girls may reflect alignment with common activity patterns within peer groups, which may favor socially interactive but lower-intensity activities. Another possible explanation relates to the availability and design of PA opportunities during the school day. Evidence from schoolyard studies suggests that certain schoolyard characteristics may support PA differently among girls and boys; for example, Andersen et al. [60] found that girls’ activity was enhanced by dance-oriented spaces and climbing structures. This could suggest that when activity options and available environments align more closely with students’ interests and are perceived as socially acceptable, they may facilitate engagement in more intensive forms of PA. Also, such activities, while socially meaningful, may not be captured as MVPA, which was the focus of the present study. Hip-worn accelerometers primarily capture locomotor activities [61] and may therefore underestimate forms of PA that are non-locomotor in nature. This may be particularly relevant when interpreting the associations observed among girls, as girls often engage more in climbing and social play in school playgrounds [62] that may be recorded as ST or light PA. Thus, as the present study did not assess the types or contexts of PA, but focused on intensity, these interpretations should be considered as tentative.

However, it is important to note that not all girls share the same PA preferences. Qualitative studies have identified groups of girls who strongly prefer competitive and physically demanding games such as soccer during school recess [63]. These girls may receive less support from their classroom peer group, as more vigorous play may not align with prevailing norms within girls’ groups, and boys may not necessarily encourage girls who participate in the same games. As children approach adolescence, girls may also face increasing social expectations to behave in a more restrained or ‘adult-like’ manner, which could reduce their participation in MVPA at school [37]. PA may therefore play a different role in social dynamics for girls versus boys, potentially influenced by gender-based social expectations or behavioral tendencies [64, 65], highlighting that positive social outcomes are not automatically a by-product of PA participation or its promotion [66].

The findings highlight the importance of considering individual social experiences in relation to in-school PA. It may be that girls with stronger peer ties engage more in sedentary or lighter intensity but socially interactive activities, or that dominant social norms do not encourage physically active behavior. However, no significant associations were found between social variables and in-school ST for either gender. These null findings align with the mixed findings reported in the recent systematic review by Kuzik et al. [20], suggesting that the social determinants of in-school ST remain unclear and may vary by context, measurement, or population.

While participation in physical activities provides opportunities for peer interaction [67], our results suggest that more positive self-reported peer relations in the classroom do not necessarily correspond to higher levels of in-school MVPA. Taken together, these findings suggest differences in the magnitude of the association between social experiences and in-school PA between girls and boys, although these should be interpreted cautiously given the lack of statistically significant gender differences. However, these findings offer insights into the complex nature of PA and peer interactions in school settings. From a practical perspective, efforts to promote PA in schools should not focus solely on increasing opportunities, but also on ensuring that these opportunities are socially inclusive. Educators should consider how classroom and recess norms, peer dynamics, and students’ sense of belonging may facilitate or hinder participation. Lastly, it should be noted that the comparison with prior research is not straightforward, because the studies regarding associations between PA and/or ST and peer-related factors have focused mainly on out-of-school activity and PA-related peer support [14, 15], and the context has an effect on the interrelations [17]. Moreover, recent reviews of school-based PA likewise provide limited insights into the role of peer relations in in-school PA [68–70], highlighting the importance of addressing this gap in the literature.

With respect to academic achievement, our results indicated that GPA was positively related to in-school ST in girls, but not among boys. The review by Kuzik et al. [20] highlights the complexity of associations between school-related sedentary behaviors and cognitive outcomes, such as academic achievement. In the review, approximately one-third of the associations examined showed favorable links between sedentary behavior and cognitive indicators [20], a pattern that aligns with the result observed for girls in the current study. Some sedentary behaviors may relate differently to learning outcomes [71], and higher in-school ST may partly reflect greater on-task behavior during lessons, which is linked to learning and could contribute to this association among girls. School days include considerable periods of ST [8], and a sedentary style of teaching and learning may be differentially associated with academic achievement among boys [72], yet contradictory evidence exists [73].

The results further suggested a positive association between MVPA and GPA among boys, whereas no significant association was observed among girls. However, this pattern did not translate into a statistically significant between-group difference and should be interpreted as a descriptive pattern. Although higher PA levels are associated with cognitive and academic benefits in prior research [4], findings on in-school PA and academic achievement have been mixed [27,74]. A recent systematic review and meta-analysis by He et al. [75] reported small but positive associations between school-based PA and academic achievement, while also emphasizing substantial variability across study designs, outcomes, and subgroups; for example, school-based PA was implemented very differently across studies, including interventions focusing on motor skill development, cognitively engaging activities, or physically active learning. Against this background, the present study focused on objectively measured in-school MVPA, and the positive association observed between in-school MVPA and GPA, at least among boys, is consistent with the overall direction of effects reported by He et al.

Although the data were collected in 2013, the findings remain relevant for contemporary school contexts. The present study focused on associations between in-school PA and ST, classroom peer ecology, and academic achievement, rather than on characterizing current patterns of students’ behaviors or experiences. While educational practices and in-school PA promotion have continued to evolve, the core structures of Finnish primary education, such as classroom-based instruction and stable peer groups, as well as the importance of peer interactions in students’ daily school experiences, largely remain unchanged. Thus, the associations observed may still provide meaningful insights for current efforts to promote physically active and socially inclusive school environments.

### Limitations

Several limitations should be considered. The cross-sectional design prevents causal conclusions, and the findings should be interpreted as associations. The data were collected in 2013, and while educational practices and technological influences have continued to evolve since then, the key constructs examined remain relevant in Finnish primary education, and the relationships observed are unlikely to depend on a particular curriculum version. Nevertheless, changes in curriculum frameworks and broader behavioral contexts should be considered when generalizing the findings to current cohorts or to other educational systems.

In the present study, classroom climate was assessed using a single-item measure, which cannot fully capture the complexity of the construct and may be more susceptible to measurement error than multi-item scales, thereby limiting the precision of estimated associations. Consequently, associations involving classroom climate should be interpreted cautiously, as the measure reflects a broad general perception of the classroom climate rather than specific dimensions of the construct. Future studies would benefit from using more comprehensive measures to capture classroom climate in greater detail. In addition, although the data were clustered within classrooms, the number of clusters relative to model complexity limited the use of cluster-robust multigroup SEM in the primary analyses. As a result, standard errors in the main models may be underestimated, and the findings, especially with respect to statistical significance, should be interpreted with caution. However, sensitivity analyses accounting for clustering yielded substantively similar results (S1 Appendix). Because the analyses focus on individual-level associations, the findings should not be interpreted as classroom-level or contextual effects.

Although accelerometers provide objective and reliable estimates of PA and ST, they are not without limitations. Hip-worn accelerometers primarily capture locomotor activities [60]. This may be relevant for interpreting the gender differences observed, as girls often engage more in climbing and social play in school playgrounds [61], which may be recorded as ST or light PA. Our study assessed only in-school MVPA and ST; commuting and leisure-time activity, which may also affect academic achievement, were not included.

Regarding academic achievement, teacher-assigned grades and GPA are commonly used indicators in educational research and show moderate correspondence with standardized achievement measures [76,77]. However, grades may also reflect classroom behavior and teacher evaluation, which should be considered when interpreting results [78].

The gender differences in the associations involving in-school MVPA warrant cautious interpretation, since formal gender comparisons did not reveal statistically significant differences. Finally, future research could benefit from person-centered approaches to identify subgroups with differing patterns of behavior and experiences. This may provide a more nuanced view of how in-school PA and ST and social dynamics co-occur within individuals. In addition, longitudinal and multilevel designs are needed to better examine how individual- and classroom-level processes jointly relate to in-school PA patterns and academic achievement. Future school-based PA intervention research may also benefit from considering social well-being outcomes.

## Conclusions

This study examined the dynamics between in-school PA and ST, classroom peer ecology, and academic achievement. In girls, more positive self-reported classroom peer relations were associated with lower levels of in-school MVPA, while greater in-school ST was linked to higher GPA. Among boys, higher in-school MVPA was related to higher GPA. Although the MVPA-related associations differed in statistical significance across groups, subsequent formal tests did not indicate statistically significant gender differences, and these subgroup-specific patterns should therefore be interpreted cautiously. Practically, the results suggest the potential value of considering how students experience in-school PA opportunities within their social environment when designing PA provision during the school day and pedagogical practices, although causal interpretations cannot be made.

This study extends previous research by focusing specifically on objectively measured in-school MVPA and ST and by examining their links with students’ perceived classroom peer ecology and academic achievement in the Finnish primary school context.

## Supporting information

S1 Appendix Gender-specific models with cluster-robust standard errors. (PDF)
