# Supplementary material for: Gendered patterns in the associations between in-school physical activity, classroom peer ecology, and academic achievement among primary school students
Source: PLoS One. 2026 Jun 22;21(6):e0351971. doi: 10.1371/journal.pone.0351971 (PMC13286205; doi:10.1371/journal.pone.0351971)
Supplement: S1 Appendix — (PDF) [file pone.0351971.s001.pdf]

S1 Appendix. Gender-specific models with cluster-robust standard errors.

Table A. Standardized estimates, confidence intervals, and p-values (in-school MVPA).

	Estimate	Lower 2.5%	Upper 2.5%	SE	*p*
**BOYS**					
Effects of peer relations on in-school MVPA	-0.07	-0.22	0.07	0.08	.327
Effects of peer relations on GPA	0.13	-0.08	0.34	0.11	.230
Effects of classroom climate on in-school MVPA	0.11	-0.03	0.24	0.07	.114
Effects of classroom climate on GPA	-0.07	-0.23	0.08	0.08	.344
Effects of in-school MVPA on GPA	0.15	0.03	0.26	0.06	.013*
Effects of age on in-school MVPA	-0.22	-0.39	-0.05	0.09	.011*
**GIRLS**					
Effects of peer relations on in-school MVPA	-0.19	-0.38	-0.00	0.10	.046*
Effects of peer relations on GPA	-0.10	-0.37	0.17	0.14	.473
Effects of classroom climate on in-school MVPA	0.09	-0.05	0.23	0.07	.215
Effects of classroom climate on GPA	0.13	-0.12	0.39	0.13	.307
Effects of in-school MVPA on GPA	0.04	-0.09	0.18	0.07	.544
Effects of age on in-school MVPA	-0.22	-0.39	-0.05	0.09	.011*

Note: MVPA = moderate-to-vigorous physical activity; GPA = grade point average; SE = standard error; $*p < .05$; Model fit for boys: $\chi2$ [24] = 51.25, p = .001; CFI = 0.91; TLI = 0.88; RMSEA = 0.07, 90% CI [0.04–0.10]; SRMR = 0.06; Model fit for girls: $\chi2$ [24] = 33.65, p = .091; CFI = 0.98; TLI = 0.97; RMSEA = 0.04, 90% CI [0.00–0.07]; SRMR = 0.04; Sensitivity analyses were conducted using gender-specific models with cluster-robust standard errors (TYPE = COMPLEX in Mplus), as cluster-robust estimation was not reliable for all parameters in the multigroup SEM.

Table B. Standardized estimates, confidence intervals, and p-values (in-school ST).

	Estimate	Lower 2.5%	Upper 2.5%	SE	*p*
**BOYS**					
Effects of peer relations on in-school ST	0.08	-0.05	0.21	0.07	.222
Effects of peer relations on GPA	0.13	-0.09	0.34	0.11	.245
Effects of classroom climate on in-school ST	-0.07	-0.23	0.09	0.08	.407
Effects of classroom climate on GPA	-0.06	-0.22	0.10	0.08	.454
Effects of in-school ST on GPA	-0.07	-0.22	0.08	0.08	.386
Effects of age on in-school ST	0.28	0.15	0.42	0.09	.000***
**GIRLS**					
Effects of peer relations on in-school ST	0.03	-0.16	0.22	0.10	.750
Effects of peer relations on GPA	-0.12	-0.37	0.14	0.13	.363
Effects of classroom climate on in-school ST	-0.04	-0.20	0.12	0.08	.620
Effects of classroom climate on GPA	0.15	-0.09	0.39	0.12	.207
Effects of in-school ST on GPA	0.15	0.03	0.28	0.06	.016*
Effects of age on in-school ST	0.35	0.22	0.48	0.07	.000***

Note: ST = sedentary time; GPA = grade point average; SE = standard error; *p < .05, ***p < .001; Model fit for boys: $\chi 2$ [24] = 53.05, p = .001; CFI = 0.91; TLI = 0.87; RMSEA = 0.07, 90% CI [0.05–0.10]; SRMR = 0.06; Model fit for girls: $\chi 2$ [24] = 31.32, p = .145; CFI = 0.99; TLI = 0.98; RMSEA = 0.03, 90% CI [0.00–0.06]; SRMR = 0.04; Sensitivity analyses were conducted using gender-specific models with cluster-robust standard errors (TYPE = COMPLEX in Mplus), as cluster-robust estimation was not reliable for all parameters in the multigroup SEM.
