# Supplementary material for: Gendered patterns in the associations between in-school physical activity, classroom peer ecology, and academic achievement among primary school students
Source: PLoS One. 2026 Jun 22;21(6):e0351971. doi: 10.1371/journal.pone.0351971 (PMC13286205; doi:10.1371/journal.pone.0351971)
Supplement: Attachment — Submitted filename: Response to Reviewers.pdf [file pone.0351971.s002.pdf]

POINT-BY-POINT RESPONSE TO REVIEWERS

Ms. Ref. No.: PONE-D-25-55757
Manuscript title: Gender-specific dynamics between in-school physical activity, classroom peer ecology, and academic achievement among primary school students

Note: All line numbers referred to in this response correspond to the revised, unmarked version of the manuscript (labeled 'Manuscript').

Journal requirements	Authors' response	Change in the manuscript
1. Please ensure that your manuscript meets PLOS ONE's style requirements, including those for file naming. The PLOS ONE style templates can be found at<br><br>https://journals.plos.org/plosone/s/file?id=wjVg/PLOSOne_formatting_sample_main_body.pdf<br><br>and<br><br>https://journals.plos.org/plosone/s/file?id=ba62/PLOSOne_formatting_sample_title_authors_affiliations.pdf	We have reviewed the templates and guidelines again and made revisions accordingly. We are happy to make additional adjustments if there are further comments.	Title page:<br><br>We adjusted the title position on the title page and changed line spacing for affiliations and reduced the font size of the title.<br><br>Manuscript:<br><br>We modified the manuscript formatting so that the first line of a paragraph starts further in.
2. Thank you for stating the following financial disclosure:<br><br>"Data collection for this study was supported by a grant (OKM/92/626/2013) from the Finnish Ministry of Education and Culture and the Juho Vainio Foundation. This manuscript was prepared as part of the SchoolWell project, which was funded by the Strategic Research Council established within the Research Council of Finland [353361 (T.H.T.) and 1252509 (K.P)]."<br><br>Please state what role the funders took in the study. If the funders had no role, please state: "The funders had no role in study design, data collection and analysis, decision to publish, or preparation of the manuscript."	The statement "The funders had no role in study design, data collection and analysis, decision to publish, or preparation of the manuscript" is correct, and we included this in the cover letter.	

If this statement is not correct you must amend it as needed.<br><br>Please include this amended Role of Funder statement in your cover letter; we will change the online submission form on your behalf.		
3. In the online submission form you indicate that your data is not available for proprietary reasons and have provided a contact point for accessing this data. Please note that your current contact point is a co-author on this manuscript. According to our Data Policy, the contact point must not be an author on the manuscript and must be an institutional contact, ideally not an individual. Please revise your data statement to a non-author institutional point of contact, such as a data access or ethics committee, and send this to us via return email. Please also include contact information for the third party organization, and please include the full citation of where the data can be found.	We have revised our data statement to a non-author institutional point of contact and sent this statement also via return email.<br><br>"Data for this study were collected as part of a larger research project called Students' physical activity and well-being. All the data are owned by Likes, Jamk University of Applied Sciences.<br><br>Restrictions apply to the availability of these data as the ethical approval received at the time of the data collection did not permit open sharing of the dataset. The data were used under a license agreement for the current study and are therefore not publicly available. Data may, however, be available from Jamk University of Applied Sciences upon reasonable request by contacting the ethical committee of Jamk (eettinentk@jamk.fi)."	
4. Please include your full ethics statement in the 'Methods' section of your manuscript file. In your statement, please include the full name of the IRB or ethics committee who approved or waived your study, as well as whether or not you obtained informed written or verbal consent. If consent was waived for your study, please include this information in your statement as well.	Our ethics statement has now been revised in the 'Methods' section. We added the date and the receiver of the ethics approval and clarified the sentence about consent.	Line numbers 143-144 (methods):<br><br>"Ethics approval for this study was obtained from the Ethics Committee of the University of Jyväskylä (11.12.2012 / T.H.T)."<br><br>Line numbers 145-146 (methods):<br><br>"..., and all participants and their guardians provided written informed consent prior to participation."

5. If the reviewer's comments include a recommendation to cite specific previously published works, please review and evaluate these publications to determine whether they are relevant and should be cited. There is no requirement to cite these works unless the editor has indicated otherwise.	We carefully reviewed the reviewers' comments and evaluated the relevance of any related literature. Where appropriate, we updated the manuscript with additional citations.	
**Additional Editor Comments:**<br><br>Major and substantive revisions are required for this manuscript. The reviewers' comments are pertinent, and the authors should address each comment thoroughly and appropriately.	We thank the editor for the evaluation of our manuscript. We have carefully considered all reviewer comments and have revised the manuscript accordingly. Each comment is addressed in detail in this point-by-point response, with corresponding changes indicated in the manuscript.	
Suggestion, question,<br>or comment from Reviewer #1	Authors' response	Change in the manuscript
6. Interesting idea of this study, my recommendations are the following: Abstract – I recommend rewriting the first sentence, mention ... for increasing physical activity (PA), it is not clear and logical, you can possibly use another verb such as: practicing or increasing the duration of practicing PA or actively involving in PA.	We appreciate the comment regarding the abstract. In response, we have revised the sentence to use a more precise verb describing participation in physical activity.	Line number 27 (abstract):<br><br>"School is an important setting for increasing students' engagement in physical activity (PA)..."
7. I recommend reviewing the keywords, certain words do not appear from the title of the study such as: Sport and exercise sciences; Social sciences. Also Academic achievement; Academic performance; in conclusion, I recommend clarifications.	We have revised the keywords by removing broad disciplinary terms and refining and shortening the list to better reflect the focus of the study.	The new keywords are listed below:<br><br>Physical activity; Sedentary behavior; Accelerometry; Academic achievement; Peer relations; Classroom climate; Primary school; Students
8. Line 20- PA cannot increase, this association of words is inappropriate, I recommend rewriting.	Please see the answer to the comment #6 and the related change made to the manuscript.	
9. Primary education in Finland – I recommend mentioning how many	We thank you for this recommendation. In Finnish primary education (grades 4–6),	Line numbers 129-130 (methods):

hours/week of PA are provided for in the curriculum in primary education.	physical education is a compulsory subject. At the time of data collection (2013), the national curriculum required approximately 90 minutes of physical education per week (2 × 45 min), as regulated by the Government Decree on the Distribution of Lesson Hours (422/2012). This minimum provision has remained largely unchanged. According to the current Government Decree (111/2023), students in grades 4–6 still receive at least two physical education lessons per week, with some local variation allowing three lessons.<br><br>In addition to physical education, students may accumulate physical activity during the school day through recess, active commuting, classroom-based physical activity, and transitions, although these are not formally regulated or quantified in the curriculum and therefore vary between schools.<br><br>We have added a concise description of this to the "Primary education in Finland" -part.	"Students in grades 4–6 received approximately 1.5 hours of curriculum-mandated PA per week through compulsory physical education (2 x 45-minute lessons)."
10. Participants and procedures – according to the information regarding the year of this study, I recommend adding clarifications regarding the difference of 12 years that have passed since 2013. I also recommend mentioning whether in those 12 years there have been changes to the curriculum structure at the primary education level. In my opinion, 12 years represents a major gap both socially and psychologically due to technological and methodological influences at the educational level. I recommend making justifications regarding the aforementioned.	We agree that the time passed since data collection warrants clarification. At the time of the study, Finnish primary education followed the National Core Curriculum for Basic Education 2004. A curriculum reform was introduced in 2014, updating the general pedagogical aims but not substantially altering the basic structure of primary education. Also, the minimum amount of curriculum-mandated physical education remained largely unchanged. Based on the national guidelines set in the core curriculum, each school district developed their local curriculum,	Line numbers 378-384 (limitations):<br><br>"The data were collected in 2013, and while educational practices and technological influences have continued to evolve since then, the key constructs examined in this study continue to be relevant in Finnish primary education. As the focus of the study is on associations between these constructs rather than on curriculum-specific effects, the observed relationships are unlikely to depend on a particular curriculum version.

	and teachers participated in developing the local curriculum. Moreover, in Finnish system, teachers are considered as highly educated professionals (with university MA degrees). Teachers have substantial autonomy in curriculum implementation, for example, they decide on pedagogical practices, choose teaching materials, etc.<br><br>Importantly, the present study does not aim to describe a specific temporal moment, but rather to examine associations between variables that remain relevant in the contemporary educational context. The key constructs examined in this study continue to exist in Finnish primary education, and we strongly believe their relationships are not dependent on a particular curriculum version.<br><br>Regarding technological influence, it is noteworthy that smartphone and social media use were already widespread by 2013, making digital media a common part of children's and adolescents' everyday lives at the time of data collection (Kakihara, 2014; Poushter, 2016).<br><br>Nevertheless, the temporal context of the data is acknowledged, and this has now been explicitly addressed in the Limitations section of the manuscript.	Nevertheless, changes in curriculum frameworks and broader behavioral contexts should be considered when generalizing the findings to current student cohorts or to other educational systems."
11. I recommend that all acronyms be mentioned descriptively under the tables.	Thank you for raising this concern. We agree that all used acronyms should be defined alongside the tables.	Table 2, line number 233: added description of the acronym SRMR (standardized root mean squared residual).<br><br>Table 3, line number 251: added descriptions of the acronyms SD (standard deviation) and ICC (intraclass correlation coefficient).

		Table 4, line number 261-262: added descriptions of the acronyms PA (physical activity), M (mean), and SD (standard deviation).<br><br>Tables 5 and 6, line numbers 276-277 and 295, respectively: added description of the acronym SE (standard error).
12. In conclusion, the study is well organized, the information has a scientific logic and is relevant.	We sincerely thank the reviewer for taking the time to evaluate our manuscript and for providing constructive feedback.	
Suggestion, question,<br>or comment from Reviewer #2	Authors' response	Change in the manuscript
13. I would like to thank to authors for this interesting study. I believe authors made a such nice introduction section with hypothesis and their problem of statement is very clear. In addition, this study had a good sample size and appropriate measurement. My question is about validity and reliability of tests and measurements. This should be reported in the study.	We sincerely thank the reviewer for taking the time to read and evaluate our manuscript.<br><br>In response to the question regarding the validity and reliability of the measures, we provide information for each study variable below and have revised the manuscript to make these aspects more explicit.<br><br>**Classroom peer relations:**<br>The classroom peer relations scale demonstrated good internal consistency (Cronbach's α = 0.81). In addition, structural validity was assessed using confirmatory factor analysis (CFA), and metric invariance was supported, with acceptable model fit indices and changes in χ^2, CFI, and RMSEA (see Table 2 in manuscript).<br><br>In addition, within the larger research project from which the present data were drawn, the test–retest reliability of the questionnaire items was evaluated among 181 students in grades 4 to 9, who completed the questionnaire twice	**Regarding classroom peer relations**, line numbers 149-151 and 152-155, respectively (methods):<br><br>"Classroom peer relations were assessed with five items capturing students' perceptions of the quality of peer interactions, including prosocial behavior, sense of belonging, and mutual respect (Table 1)."<br><br>"The scale showed good internal consistency (α = .81). Structural validity was supported by confirmatory factor analysis (Table 2). Test–retest reliability was assessed among 181 students in grades 4–9 using a two-week interval as described earlier [40]. The scale showed good repeatability (ICC = 0.81, 95% CI: 0.76–0.86), with single-item ICCs ranging from 0.66 to 0.71."<br><br>**Regarding classroom climate**, line numbers 162-163 (methods) and 385-389 (limitations):

	with a two-week interval (for study setting, see Pirnes et al. 2019). Questions for peer relations had good repeatability with the intraclass correlation coefficient (ICC) for the scale being 0.81 (95%CI 0.76-0.86) and for single items between 0.66-0.71.<br><br>**Classroom climate:**<br>Classroom climate was assessed using a single item, and we acknowledge the limitations associated with this approach. However, the item demonstrated good test–retest reliability, with an intraclass correlation coefficient (ICC) of 0.73 (95% CI: 0.66–0.79). Nevertheless, the limitations of using a single-item measure have now been explicitly acknowledged in the manuscript.<br><br>**Physical activity and sedentary time using accelerometry:**<br>Waist-mounted accelerometers (such as ActiGraph GT3X+ and wGT3X+) are widely used in physical activity research (Cain et al. 2013), and they have been shown to provide valid and reliable estimates of children's physical activity and sedentary time (Barreira et al. 2015; Janssen et al. 2013; Phillips et al. 2021).<br><br>**GPA:**<br>Teacher-assigned grades and GPA are widely used indicators of academic achievement in educational research (Costa et al., 2024). Prior research shows moderate to strong associations between grades and standardized achievement tests, supporting their criterion validity; for example, in Finland, the correspondence between PISA scores and teacher-rated grades is relatively high (Pulkkinen & Rautopuro, 2022).	"The item demonstrated good test–retest reliability (ICC = 0.73, 95% CI: 0.66–0.79)."<br><br>"Classroom climate was assessed using a single-item measure, which may be more susceptible to measurement error than multi-item scales and therefore limit the precision of estimated associations. As a result, associations involving classroom climate should be interpreted with caution. Future studies would benefit from using more comprehensive measures to capture classroom climate in greater detail."<br><br>**Regarding PA and ST**, line numbers 167-168 (methods):<br><br>"Accelerometers have been shown to provide valid and reliable estimates of children's PA and ST [42-43."<br><br>**Regarding GPA**, line numbers 402-405 (limitations):<br><br>"Regarding academic achievement, teacher-assigned grades and GPA are commonly used indicators in educational research and show moderate correspondence with standardized achievement measures [75-76]. However, grades may also reflect classroom behavior and teacher evaluation, which should be considered when interpreting results [77]."

	GPA has also demonstrated strong predictive validity for students' later educational trajectories (Steinmayr et al., 2014) and later educational success (Cliffordson, 2008). However, grades reflect not only academic competencies but also classroom behavior and teacher evaluation. Moreover, gender differences in grading (typically favoring girls) have been reported (Thorsen & Cliffordson, 2012). These should be considered when interpreting research results on GPAs, and we have now acknowledged this in the manuscript.	
14. Moreover, this study needs a strong discussion and conclusion sections with up to date literature review (2023 and above) with practical suggestions for the future. I look forward to seeing edited version of this timely and interesting manuscript. Thank you.	We thank the reviewer for these suggestions. We have revised and expanded the Discussion section to include relevant, recent literature where available and applicable.<br><br>To our best knowledge, there are no review articles that combine all constructs examined in the manuscript - in-school PA/ST, peer ecology, and academic achievement.<br><br>Regarding recent systematic reviews potentially addressing in-school PA/ST and peer relations, the review and meta-analyses by Moeller et al. (2024) primarily focus on school-based PA intervention characteristics and implementation factors rather than peer-related processes. A meta-synthesis on peer effects on physical exercise (Chen et al., 2026) addresses peer influences but is not specific to in-school PA. A review focusing on PA during school recess (Zhu et al., 2025) includes only one study that examined peer relations, which is already included in the present manuscript. Similarly, a review of	Line numbers 364-371 (discussion):<br><br>"A recent systematic review and meta-analysis by He et al. [70] reported small but positive associations between school-based PA and academic achievement, while also emphasizing substantial variability across study designs, outcomes, and subgroups. For example, school-based PA was implemented very differently across studies, including interventions focusing on motor skill development, cognitively engaging activities, or physically active learning. Our study focused on examining associations with the amount of objectively measured in-school MVPA. The positive association observed between in-school MVPA and GPA among boys is consistent with the overall direction of effects reported by He et al."

	school environmental correlates of PA (Wang et al., 2025) assesses social aspects of the school environment but does not address peer-related factors. Taken together, these recent reviews highlight a gap in the literature regarding the role of peer relations in relation to objectively measured in-school PA and ST. We have revised the Discussion to clarify this gap and to better position the present study within the current evidence base.<br><br>To our best knowledge, the present manuscript includes the most recent and most relevant review articles examining (a) peer-related factors in relation to academic achievement and (b) ST and academic achievement, although these were published slightly prior to 2023.<br><br>Regarding the practical suggestions, we have expanded the Discussion, Limitations, and Conclusions sections to more explicitly address practical implications and future research directions. We now highlight the potential value of considering students' social experiences when designing in-school physical activity opportunities and pedagogical practices, while maintaining non-causal interpretation of the findings. In addition, we have added research-oriented recommendations.	Line numbers 322-326 (discussion):<br><br>"Stearns et al. [59] also suggest that among girls, school friends tend to have similar PA levels, rather than friendship consistently leading to higher PA levels. Thus, similarities in PA patterns may attract and sustain friendships. Accordingly, stronger peer relations among girls may reflect alignment with common activity patterns within peer groups, which may favor socially interactive but lower-intensity activities."<br><br>Line numbers 351-353 (discussion):<br><br>"Moreover, recent reviews of school-based PA likewise provide limited insights into the role of peer relations in PA during the school day [65-67], highlighting the importance of addressing this gap in the literature."<br><br>Line numbers 409-414 (limitations):<br><br>"Finally, future research could benefit from person-centered approaches to identify subgroups with differing patterns of behavior and experiences. This may provide a more nuanced view of how in-school PA and ST and social dynamics co-occur within individuals. In addition, longitudinal and multilevel designs are needed to better examine how individual- and classroom-level processes jointly relate to in-school PA patterns and academic

		outcomes. Future school-based PA intervention research may also benefit from considering social well-being outcomes.”
Suggestion, question,<br>or comment from Reviewer #3	Authors’ response	Change in the manuscript
15. Dear Authors,<br>Thank you for submitting your manuscript. The topic is potentially relevant; however, in its current form the study has several fundamental issues that substantially limit the credibility, reproducibility, and interpretability of the findings. For these reasons, I recommend rejection.	We thank the reviewer for their careful reading of the manuscript and for raising these concerns. Below, we respond to each point in detail and indicate the corresponding changes made to the manuscript.	
16. Data availability and compliance with PLOS Data Policy:<br><br>PLOS requires that all data underlying the reported findings be made fully available without restriction. In the current submission, the underlying dataset is not clearly provided in an unrestricted, accessible form, and the description suggests that access may depend on requesting data from the authors/third parties. This does not meet the expected standard for transparency and reproducibility unless a clearly justified exception is documented and an alternative compliant solution is provided.	Thank you for raising this important point. We have revised the Data Availability Statement to ensure compliance with PLOS Data Policy. The revised statement clarifies data ownership, explains that open sharing is restricted due to the conditions of the ethical approval and the data ownership, and specifies that access requests are handled through a non-author institutional contact at the data-owning organization. All data access requests are evaluated by the institution in accordance with ethical guidelines.<br><br>Please see also the response #3 to the Editor related to journal requirements.	
17. Major statistical validity concern clustered (hierarchical) data not appropriately modeled:<br><br>The data structure is clustered (e.g., students nested within classes/schools). While the manuscript acknowledges intraclass correlation (ICC), it also states that the final modeling did not	We explored cluster-robust modeling to account for the nested data structure. However, given the number of classrooms relative to the complexity of the multigroup SEM, cluster-robust estimation was not reliable in the multigroup models.<br><br>Because gender differences were a central focus of the study, we	Line numbers 201-208 (methods):<br><br>“Multigroup cluster-robust SEM models were explored to account for the nesting of students within classrooms. However, given the number of classrooms relative to the complexity of the multigroup

incorporate clustering due to technical limitations. This is a serious methodological flaw because ignoring the hierarchical structure can bias standard errors and p-values, inflating statistical significance and undermining inferential validity. This issue affects the core conclusions and cannot be treated as a minor limitation.	therefore retained the single-level multigroup SEM as the primary analytic framework and interpreted these results explicitly as individual-level associations. To evaluate the robustness of the findings, we conducted sensitivity analyses using cluster-robust estimation in gender-specific models. These analyses yielded substantively similar patterns of results, with the key associations remaining statistically significant (please see S1 Appendix for results).<br><br>We have revised the manuscript to clarify this and to acknowledge the implications for interpreting the results.	SEM, cluster-robust estimation was not reliable for all parameters. Because gender differences were a focus of the study, the primary analyses were conducted using single-level multigroup SEM and are interpreted as individual-level associations. To assess the robustness of the findings, sensitivity analyses were conducted by estimating gender-specific models using cluster-robust standard errors (TYPE = COMPLEX in Mplus). These analyses yielded substantively similar patterns of results, with the key associations remaining statistically significant (S1 Appendix).”<br><br>Line numbers 389-395: (limitations):<br><br>“In addition, although the data were clustered within classrooms, the number of clusters relative to model complexity limited the use of cluster-robust multigroup SEM in the primary analyses. As a result, standard errors in the main models may be underestimated, and the findings, especially with respect to statistical significance, should be interpreted with caution. However, sensitivity analyses accounting for clustering yielded substantively similar results. Because the analyses focus on individual-level associations, the findings should not be interpreted as classroom-level or contextual effects.”<br><br>**Also (throughout the manuscript):**

		Language implying causal or classroom-level effects was revised throughout the manuscript. At the same time, we aimed to retain practical relevance by framing implications cautiously, without overinterpretation.
18. Limited explanatory value and weak practical significance:<br><br>The reported models appear to have very low explanatory power (small R^2 values) with only a small number of statistically significant pathways. As a result, the added scientific value is limited and the manuscript risks overemphasizing marginal effects that may not be robust.	We acknowledge that the proportion of explained variance in the models is modest. However, the primary aim of the present study was to examine associations between in-school PA, ST, perceived classroom peer relations and classroom climate, and academic achievement, rather than to maximize explained variance or to develop a predictive model.<br><br>In-school PA and ST, and academic achievement are multifactorial outcomes influenced by a wide range of biological, behavioral, social, and contextual factors, many of which were beyond the scope of the present study. Accordingly, modest R^2 values are common in studies examining associations between PA and school- and peer-related outcomes, and such values are not very uncommon in the existing literature (e.g., Haapala et al., 2014; Milošević & Tubić, 2018). In addition, unfortunately R^2 values are not consistently reported across studies in this literature, which makes direct comparisons of explanatory power challenging (e.g., Arufe-Giráldez et al., 2019; Lodewyk et al., 2019).<br><br>However, to strengthen the models, we included age as a covariate in the analyses, given its well-established inverse association with PA and positive association with ST in children and adolescents.	Line numbers 238-241 (methods):<br><br>"Age was included as a covariate in both models, with regression paths specified from age to in-school MVPA and in-school ST, given the well-established associations between age and children's PA and sedentary behavior [57]."<br><br>Line numbers 278-281 (results):<br><br>"The explained overall variance was modest across outcomes based on R^2 estimates. The model accounted for 5.6% of the variance in in-school MVPA among boys ($p = .031$) and 7.2% among girls ($p = .023$), whereas the variance explained in GPA was 3.2% ($p = .238$) and 1.5% ($p = .462$), respectively."<br><br>Line numbers 296-299 (results):<br><br>"As in the model with in-school MVPA, the explained variance was modest across outcomes. The model accounted for 8.4% of the variance in in-school ST among boys ($p = .010$) and 12.2% among girls ($p < .001$), whereas the variance explained in GPA was 1.5% ($p = .456$) for

	Importantly, adjusting for age increased the explained variance in in-school MVPA and ST and did not attenuate the key associations observed in the previous models, supporting the robustness of the findings. We have revised the manuscript accordingly.	boys and 3.8% (p = .223) for girls."<br><br>Also, numerical changes have been made to the Results section based on the new analyses that includes age as a covariate for in-school MVPA and ST (see also tables and figures).
19. Study design constraints and temporal relevance:<br><br>The cross-sectional design does not support causal inference, yet parts of the discussion and implications drift toward causal language and strong recommendations. In addition, the dataset is relatively old, and the manuscript does not sufficiently justify the contemporary relevance and generalizability of the findings to current educational/behavioral contexts.	We agree that the cross-sectional design does not permit causal inference, and we have revised the discussion and conclusion sections to remove causal language and strong recommendations. In addition, we have highlighted this in the Limitations.<br><br>Regarding the temporal context of the data, we agree that the manuscript needs better consideration of contemporary relevance and generalizability.<br><br>At the educational context, data collection took place under the Finnish National Core Curriculum for Basic Education 2004. Although a curriculum reform was introduced in 2014, this reform did not substantially alter the core structure of primary education, including the organization of the school day, minimum physical education requirements, or the high degree of teacher autonomy.<br><br>The present study does not aim to describe a specific temporal moment, but rather to examine associations between variables that remain relevant in the contemporary educational context. The key constructs examined in this study continue to exist in Finnish primary education, and we believe there is no strong reason to assume that the relationships between these	Line numbers 377-378 (limitations):<br><br>"The cross-sectional design prevents causal conclusions, and the findings should be interpreted as associations."<br><br>Line numbers 378-384 (limitations):<br><br>"The data were collected in 2013, and while educational practices and technological influences have continued to evolve since then, the key constructs examined in this study continue to be relevant in Finnish primary education. As the focus of the study is on associations between these constructs rather than on curriculum-specific effects, the observed relationships are unlikely to depend on a particular curriculum version. Nevertheless, changes in curriculum frameworks and broader behavioral contexts should be considered when generalizing the findings to current student cohorts or to other educational systems."

	constructs are specific to a particular historical period or curriculum version.<br><br>However, we also acknowledge technological developments that could affect the behavioral context. Notably, smartphone and social media use were already widespread by 2013, making digital media a common part of children's everyday lives at the time of data collection (Kakihara, 2014; Poushter, 2016).<br><br>Nevertheless, the temporal context of the dataset and its potential implications are now explicitly acknowledged in the Limitations section of the manuscript.	
20. Over-interpretation of non-intuitive associations without adequate robustness checks:<br><br>Some reported associations are not intuitive (e.g., unexpected directions of relationships) and are explained largely through post-hoc speculation. The manuscript would require stronger triangulation, sensitivity analyses, and/or alternative model specifications to support such interpretations.	We note that the study was guided by research questions rather than directional hypotheses, as this work addresses a relatively novel combination of in-school physical activity and sedentary time with social and academic variables. As such, the analyses were intended to be exploratory and descriptive of patterns.<br><br>To address concerns about robustness, we conducted additional sensitivity analyses using cluster-robust estimation in gender-specific models. These analyses yielded substantively similar patterns of results. Also, when age was added as a predictor to in-school MVPA and ST, it did not attenuate the prior findings on paths, even though age is known to be a significant predictor of children's PA and ST levels.<br><br>We revised the Discussion and Conclusions to limit speculative explanations and interpretations. The Limitations section was expanded to acknowledge design	Please see the changes referred to in parts #17, #18, #19, and #21.

	and measurement constraints and to outline directions for future research.	
21. Measurement limitations for key constructs:<br><br>Using very brief or single-item measures for important constructs (e.g., classroom climate) raises concerns about measurement error and construct validity especially when these measures are used in complex models (e.g., SEM). This further weakens confidence in the reported pathways.	We agree that the use of a single-item measure for classroom climate introduces a greater risk for measurement error and limits the precision of its pathway estimates. The classroom climate item was part of the original questionnaire of a larger longitudinal school-based project, where the length of the survey required the use of brief measures for some constructs.<br><br>The classroom climate item demonstrated good test–retest reliability and showed positive correlations with the latent classroom peer relations construct, consistent with theoretical expectations.<br><br>Single-item measures are more susceptible to measurement error, which is expected to attenuate associations with outcomes rather than inflate them. Consistent with this, classroom climate did not show a significant association with the outcomes. Only peer relations showed a significant association with in-school MVPA, which is consistent with the possibility that peer relations represent a more behaviorally proximal social mechanism influencing students' PA during the school day. We have avoided any strong interpretations of the classroom climate pathway and have revised the manuscript to explicitly acknowledge the limitations of single-item measurement.<br><br>Finally, single-item indicators of contextual or psychological constructs have been used in prior research when theoretically justified	Line numbers 385-389 (limitations):<br><br>"Classroom climate was assessed using a single-item measure, which may be more susceptible to measurement error than multi-item scales and therefore limit the precision of estimated associations. As a result, associations involving classroom climate should be interpreted with caution. Future studies would benefit from using more comprehensive multi-item measures to capture classroom climate in greater detail."

	and practically constrained (e.g., Haapala et al. 2014; Eddy et al., 2019), although we emphasize that future studies should employ more comprehensive assessments.	
22. Given the combination of (i) data availability concerns, and (ii) a critical modeling limitation related to clustered data alongside low explanatory power and interpretive overreach I do not believe the manuscript is suitable for publication in its current form.	We appreciate the reviewer's careful evaluation of the manuscript and acknowledge that the concerns raised are important. In response, we have undertaken substantial revisions to address each of the issues highlighted. We hope that the revised version adequately addresses the reviewer's concerns and clarifies the scope and contribution of the study.	

References cited in the response to reviewers:

Arufe-Giráldez V, Zurita-Ortega F, Padial-Ruz R, Castro-Sánchez M. Association between level of empathy, attitude towards physical education and victimization in adolescents: A multi-group structural equation analysis. Int J Environ Res Public Health. 2019;16(13):2360. doi:10.3390/ijerph16132360.

Barreira TV, Schuna JM, Tudor-Locke C, Chaput JP, Church TS, Fogelholm M, et al. Reliability of accelerometer-determined physical activity and sedentary behavior in school-aged children: A 12-country study. Int J Obes Suppl. 2015;5(Suppl 2):S29-S35. doi:10.1038/ijosup.2015.16.

Cain KL, Sallis JF, Conway TL, Van Dyck D, Calhoon L. Using accelerometers in youth physical activity studies: A review of methods. J Phys Act Health. 2013;10(3):437-450. doi:10.1123/jpah.10.3.437.

Chen K, Chen C, Yang J, Li Z. The impact of peer effects on adolescents' physical exercise: A meta-synthesis study. Front Psychol. 2026;16:1748992. doi:10.3389/fpsyg.2025.1748992.

Cliffordson C. Differential prediction of study success across academic programs in the Swedish context: The validity of grades and tests as selection instruments for higher education. Educ Assess. 2008;13:56-75. doi:10.1080/10627190801968240.

Costa A, Moreira D, Casanova J, Azevedo Â, Gonçalves A, Oliveira Í, et al. Determinants of academic achievement from middle to secondary school education: A systematic review. Soc Psychol Educ. 2024;27:3533-3572. doi:10.1007/s11218-024-09941-z.

Eddy CL, Herman KC, Reinke WM. Single-item teacher stress and coping measures: Concurrent and predictive validity and sensitivity to change. J Sch Psychol. 2019;76:17-32. doi:10.1016/j.jsp.2019.05.001.

Haapala H, Hirvensalo M, Laine K, Laakso L, Hakonen H, Kankaanpää A, et al. Recess physical activity and school-related social factors in Finnish primary and lower secondary schools: Cross-sectional associations. BMC Public Health. 2014;14(1):1114. doi:10.1186/1471-2458-14-1114.

Janssen X, Cliff DP, Reilly JJ, Hinkley T, Jones RA, et al. Predictive validity and classification accuracy of ActiGraph energy expenditure equations and cut-points in young children. PLOS ONE. 2013;8(11):e79124. doi:10.1371/journal.pone.0079124.

Kakihara M. Grasping a global view of smartphone diffusion: An analysis from a global smartphone study. In: Proceedings of the 11th International Conference on Mobile Business; 2014.

Lodewyk K, McNamara L, Sullivan P. Associations between elementary students' victimization, peer belonging, affect, physical activity, and enjoyment by gender during recess. Can J Sch Psychol. 2019;35:0829573519856814. doi:10.1177/0829573519856814.

Milošević Ž, Tubić T. Social support and physical activity level of elementary school students. Fiz Kult. 2018;72(1):29-36. doi:10.5937/fizkul1801029M.

Moeller NC, Oestergaard L, Rasmussen MGB, Schmidt-Persson J, Larsen KT, Juhl CB. How to get children moving? The effectiveness of school-based interventions promoting physical activity in children and adolescents: A systematic review and meta-analysis of randomized controlled and controlled studies. Health Place. 2024;89:103333. doi:10.1016/j.healthplace.2024.103333.

Phillips SM, Summerbell C, Hobbs M, Hesketh KR, Saxena S, Muir C, et al. Systematic review of the validity, reliability, and feasibility of measurement tools used to assess the physical activity and sedentary behaviour of pre-school aged children. Int J Behav Nutr Phys Act. 2021;18(1):141. doi:10.1186/s12966-021-01132-9.

Pirnes K, Kallio J, Siekkinen K, Hakonen H, Häkkinen A, Tammelin T. Test-retest repeatability of questionnaire for pain symptoms for school children aged 10–15 years. Scand J Pain. 2019;19(3):575-582. doi:10.1515/sjpain-2018-0338.

Poushter J. Smartphone ownership and internet usage continues to climb in emerging economies. Pew Res Cent. 2016;22:1-44.
Pulkkinen J, Rautopuro J. The correspondence between PISA performance and school achievement in Finland. Int J Educ Res. 2022;114:102000. doi:10.1016/j.ijer.2022.102000.

Steinmayr R, Meiǹer A, Weidinger AF, Wirthwein L. Academic achievement. Oxford: Oxford University Press; 2014. doi:10.1093/OBO/9780199756810-0108.

Thorsen C, Cliffordson C. Teachers' grade assignment and the predictive validity of criterion-referenced grades. Educ Res Eval. 2012;18(2):153-172. doi:10.1080/13803611.2012.659929.

Wang H, Pang J, Yang X, Jia Y, Huang X, Yu L, et al. School-based environment and physical activity in adolescents: A systematic review and meta-analysis. Prev Med. 2025;191:108221. doi:10.1016/j.ypmed.2025.108221.

Zhu K, Yin X, Liu Y, Qin G, Jia J, Xie Z, et al. Correlates of physical activity and sedentary behavior in children and adolescents during school recess: A systematic review and meta-analysis. BMC Public Health. 2025;25(1):2662. doi:10.1186/s12889-025-23948-x.

References added to the manuscript:

Atkin AJ, Sherar LB, Ekelund U, Hansen BH, Andersen LB, Anderssen S, et al. Age-related change in children’s physical activity and sedentary time: The International Children’s Accelerometry Database (ICAD). PLOS ONE. 2025;20(9):e0327394. doi:10.1371/journal.pone.0327394.

Barreira TV, Schuna JM, Tudor-Locke C, Chaput JP, Church TS, Fogelholm M, et al. Reliability of accelerometer-determined physical activity and sedentary behavior in school-aged children: A 12-country study. Int J Obes Suppl. 2015;5(Suppl 2):S29-S35. doi:10.1038/ijosup.2015.16.

Costa A, Moreira D, Casanova J, Azevedo Â, Gonçalves A, Oliveira Í, et al. Determinants of academic achievement from middle to secondary school education: A systematic review. Soc Psychol Educ. 2024;27:3533-3572. doi:10.1007/s11218-024-09941-z.

He H, Yang Y, Sun J, Wang F, Zhang W, Zhu F. Effects of school-based physical activity on academic achievement in children and adolescents: A systematic review and meta-analysis. Front Public Health. 2025;13:1651883. doi:10.3389/fpubh.2025.1651883.

Moeller NC, Oestergaard L, Rasmussen MGB, Schmidt-Persson J, Larsen KT, Juhl CB. How to get children moving? The effectiveness of school-based interventions promoting physical activity in children and adolescents: A systematic review and meta-analysis of randomized controlled and controlled studies. Health Place. 2024;89:103333. doi:10.1016/j.healthplace.2024.103333.

Phillips SM, Summerbell C, Hobbs M, Hesketh KR, Saxena S, Muir C, et al. Systematic review of the validity, reliability, and feasibility of measurement tools used to assess the physical activity and sedentary behaviour of pre-school aged children. Int J Behav Nutr Phys Act. 2021;18(1):141. doi:10.1186/s12966-021-01132-9.

Pirnes K, Kallio J, Siekkinen K, Hakonen H, Häkkinen A, Tammelin T. Test-retest repeatability of questionnaire for pain symptoms for school children aged 10–15 years. Scand J Pain. 2019;19(3):575-582. doi:10.1515/sjpain-2018-0338.

Pulkkinen J, Rautopuro J. The correspondence between PISA performance and school achievement in Finland. Int J Educ Res. 2022;114:102000. doi:10.1016/j.ijer.2022.102000.

Stearns JA, Godley J, Veugelers PJ, Ekwaru JP, Bastian K, Wu B, et al. Associations of friendship and children's physical activity during and outside of school: A social network study. SSM Popul Health. 2019;7:100308. doi:10.1016/j.ssmph.2018.10.008.

Thorsen C, Cliffordson C. Teachers' grade assignment and the predictive validity of criterion-referenced grades. Educ Res Eval. 2012;18(2):153-172. doi:10.1080/13803611.2012.659929.

Wang H, Pang J, Yang X, Jia Y, Huang X, Yu L, et al. School-based environment and physical activity in adolescents: A systematic review and meta-analysis. Prev Med. 2025;191:108221. doi:10.1016/j.ypmed.2025.108221.

Zhu K, Yin X, Liu Y, Qin G, Jia J, Xie Z, et al. Correlates of physical activity and sedentary behavior in children and adolescents during school recess: A systematic review and meta-analysis. BMC Public Health. 2025;25(1):2662. doi:10.1186/s12889-025-23948-x.
