# Supplementary material for: Gendered patterns in the associations between in-school physical activity, classroom peer ecology, and academic achievement among primary school students
Source: PLoS One. 2026 Jun 22;21(6):e0351971. doi: 10.1371/journal.pone.0351971 (PMC13286205; doi:10.1371/journal.pone.0351971)
Supplement: Attachment — Submitted filename: Response_to_Reviewers_auresp_2.pdf [file pone.0351971.s003.pdf]

POINT-BY-POINT RESPONSE TO REVIEWERS

Ms. Ref. No.: PONE-D-25-55757R2
Manuscript title: Gender-specific dynamics between in-school physical activity, classroom peer ecology, and academic achievement among primary school students

Note: All line numbers referred to in this response correspond to the revised, unmarked version of the manuscript (labeled ‘Manuscript’).

Journal requirements	Authors’ response	Change in the manuscript
1. If the reviewer's comments include a recommendation to cite specific previously published works, please review and evaluate these publications to determine whether they are relevant and should be cited. There is no requirement to cite these works unless the editor has indicated otherwise.	We carefully reviewed the reviewers’ comments; however, no specific citations were suggested.	
**Additional Editor Comments:**<br><br>2. Please ensure that all reviewers’ comments are addressed clearly and comprehensively, where appropriate.<br><br>Kind regards!	We thank the editor for the evaluation of our manuscript. We have carefully considered all reviewer comments and have revised the manuscript accordingly. Each comment is addressed in detail in this point-by-point response, with corresponding changes indicated in the manuscript.	
Suggestion, question,<br>or comment from Reviewer #1	Authors’ response	Change in the manuscript
3. No comments. The authors have revised the article in accordance with the above recommendations. I believe that in its current form the article is ready for the journal.	We sincerely thank the reviewer for taking the time to evaluate our manuscript and for acknowledging that the manuscript has been improved in accordance with the recommendations.	
Suggestion, question,<br>or comment from Reviewer #2	Authors’ response	Change in the manuscript
4. I believe the current edited version this manuscript is acceptable. All my questions answered in detail. Thank you.	We sincerely thank the reviewer for taking the time to evaluate our manuscript and for confirming that all previous concerns have been adequately addressed.	

Suggestion, question, or comment from Reviewer #4	Authors' response	Change in the manuscript
5. This is an interesting manuscript with a meaningful contribution using objectively measured in-school movement behaviours and academic achievement in primary school students. That combination is valuable, especially because the study uses accelerometer-based school-time MVPA/ST rather than relying entirely on self-report, and because it takes the school/classroom context seriously rather than treating activity as a purely individual behaviour. The research questions are clear and the manuscript is generally well organized. Furthermore, it is very sound science using objectively measured in-school MVPA and ST, the methods are clear and the modeling is well thought out. Most importantly, it has a practical resonance for educators.	We sincerely thank the reviewer for their comprehensive and insightful evaluation of the manuscript and for their positive comments on the study's contribution, methodology, and practical relevance.<br><br>Below, we respond to each of the comments and concerns in detail and indicate the corresponding changes made to the manuscript.	
6. Major concerns:<br><br>The manuscript repeatedly emphasizes "gendered" or "gender-specific" patterns in the abstract, discussion, and conclusion. But in the MVPA model, the authors explicitly report that constraining the significant paths to equality across genders did not significantly worsen fit, meaning those associations did not significantly differ by gender. Yes, the authors did disclose in the" limitations, but the big picture is that the paper cannot say that the MVPA associations did not significantly differ by gender! Interpretation of gender has to be consistent across Results, Discussion, Conclusion...	We thank for this careful observation. As noted in the manuscript, constraining the paths to equality across genders in the MVPA-model did not significantly worsen model fit, indicating that the associations did not differ statistically by gender.<br><br>It is worth noting that the formal comparison of paths across groups represents a very strict test of subgroup differences, and more subtle differences in parameter estimates may not reach statistical significance, and the differences in the magnitude of the estimates and p-values in multigroup SEM may still suggest meaningful gender-specific patterns.<br><br>However, we agree that interpretations of gender differences should be more consistent with the formal statistical tests throughout	**Abstract,** line numbers 39–43:<br><br>"Associations were investigated through multigroup structural equation modeling analyses. In girls, positive classroom peer relations were associated with lower in-school MVPA, while greater in-school ST was linked to higher GPA. Among boys, higher in-school MVPA was related to higher GPA. Although subsequent formal tests indicated that the MVPA-related associations did not statistically differ by gender, the observed patterns varied in magnitude between boys and girls."<br><br>**Discussion,** line numbers 309–315:<br><br>"The findings identified some gender-specific patterns in the

	the manuscript. Accordingly, we have revised the Abstract, Discussion, and Conclusion sections to ensure consistent and more cautious wording.	dynamics between the perceived peer ecology, in-school MVPA, in-school ST, and academic achievement.<br><br>In the current study, more positive perceptions of classroom peer relations were significantly associated with less time spent in MVPA during school hours among girls, but not among boys; however, subsequent formal tests indicated that this associations did not significantly differ by gender."<br><br>and 321–323:<br><br>"This somewhat unexpected finding suggests a potential inverse association between self-reported peer relations in the classroom and engagement in more intense forms of PA at least among girls at school."<br><br>and 365–368:<br><br>"Taken together, these findings suggest that the associations between social experiences and in-school PA may vary in magnitude between boys and girls, although these differences should be interpreted cautiously given the lack of statistically significant gender differences."<br><br>**Conclusions,** line numbers 441–447:<br><br>"This study examined the dynamics between in-school PA and ST, classroom peer ecology, and academic achievement, and the patterns in these associations by gender. In girls, more positive self-reported classroom peer

		relations were associated with lower levels of in-school MVPA, while greater in-school ST was linked to higher GPA. Among boys, higher in-school MVPA was related to higher GPA. Although subsequent formal tests indicated that the MVPA-related associations did not appear to differ statistically by gender, the observed patterns varied in magnitude between boys and girls.”
7. The study is cross-sectional, the data were collected in 2013, and the explained variance in GPA is quite modest. The models explain only a small proportion of GPA variance, and the authors themselves note that the findings should be interpreted as individual-level associations, not classroom-level effects. However, the manuscript sometimes sounds close to recommending how schools should design physically active school days based on these findings. The Reviewer believes the evidence base here is not strong enough for broad pedagogical recommendations.	We fully agree that the findings should not be interpreted as evidence supporting causal conclusions. We note that this work addresses a relatively novel combination of in-school PA and ST with social and academic variables. GPA, as well as in-school PA and ST, are multifactorial outcomes influenced by a wide range of factors, many of which were beyond the scope of the present study. Accordingly, modest R^2 values were expected. We have avoided making overly broad pedagogical recommendations, while still retaining practical relevance by framing implications cautiously and avoiding overinterpretation.<br><br>We carefully revised the manuscript to further soften language, especially regarding GPA (where the explained variance is smallest), throughout the Discussion and Conclusion sections, removing any wording that could be interpreted as prescriptive or causal.	**Discussion**, line numbers 396–399:<br><br>“School days include considerable amounts of ST [8], and a sedentary style of teaching and learning may be differentially associated with academic outcomes among boys [71], yet contradictory evidence exists [72]. Overall, the results suggest differences in patterns by gender in how in-school PA and ST are linked with academic achievement.”<br><br>**Conclusion**, line numbers 447–449:<br><br>“Practically, the results suggest the potential value of considering how students experience in-school PA opportunities within their social environment when designing PA provision during the school day and pedagogical practices, although causal interpretations cannot be made.”
8. The negative association between peer relations and girls' in-school MVPA is interesting, but much of the explanation offered in the discussion goes beyond what the data can	In response to this helpful suggestion, we have revised the Discussion. Alongside the original interpretation, we now consider measurement-related explanations,	**Discussion**, line numbers 332–345:<br><br>“Another possible explanation relates to the availability and

support. The manuscript suggests girls may avoid vigorous activity due to social discomfort or exclusion, or that peer norms may favour lower-intensity activity. These are plausible ideas, but they were not measured directly. Consider offering 2-3 alternative explanations with equal weight, including measurement limitations of hip-worn accelerometers and the possibility that socially valued activity for girls may not register well as MVPA.	including limitations of hip-worn accelerometers, which primarily capture locomotor activity and may not fully reflect socially oriented or non-locomotor movement. We now also more explicitly note that socially meaningful activities common among girls may therefore be underrepresented in MVPA estimates. Evidence suggesting that the availability of opportunities and environments may shape engagement in PA is also discussed. We further clarify that these interpretations are tentative, as activity type and context were not directly assessed.	design of PA opportunities during the school day. Evidence from schoolyard studies suggests that different types of features may support PA in gender-specific ways; for example, Andersen et al. [60] found that girls' activity was associated with features such as dance-oriented spaces and climbing structures. This could suggest that when activity options and available environments align more closely with students' interests and are perceived as socially acceptable, they may facilitate engagement in more intensive forms of PA. Also, these types of activities, while socially meaningful, may not be captured as MVPA, which was the focus of the present study. Hip-worn accelerometers primarily capture locomotor activities [61] and may therefore underestimate forms of PA that are non-locomotor in nature. This may be particularly relevant when interpreting the observed associations among girls, as girls often engage more in climbing and social play in school playgrounds [62], which may be recorded as ST or light PA. Thus, as the present study did not assess the types or contexts of PA, but focused on intensity, these interpretations should be considered as tentative."
9. "Classroom climate" is a rich construct that usually involves emotional safety, norms, teacher support, belonging, participation structure, and sometimes behaviour management or instructional organization. A single item on how students experience the class climate	We thank the reviewer for this important comment. We agree that classroom climate is a multifaceted construct, and that a single-item measure cannot fully capture the complexity.	**Limitations**, line numbers 409–413:<br><br>"In the present study, classroom climate was assessed using a single-item measure, which cannot fully capture the complexity of the construct and

is unlikely to capture that complexity. Explain why this still adds value despite being conceptually thin and avoid overinterpreting null findings for classroom climate.	The classroom climate item was part of the original questionnaire of a larger longitudinal school-based project, where practical constraints required the use of brief measures for some constructs.<br><br>Our study also includes a multi-item measure of classroom peer relations. In this context, the single-item classroom climate measure adds value to provide a broader indicator of students' general perceptions of the classroom environment, beyond peer relations. So despite its conceptual simplicity, its inclusion provides added value by capturing a more general classroom-level perception that complements the more detailed peer relations measure, thereby broadening the contextual perspective.<br><br>Single-item indicators of contextual or psychological constructs have been used in prior research (e.g., Haapala et al. 2014; Song et al. 2023), although we emphasize that future studies would benefit from employing more comprehensive assessments.<br><br>Importantly, the item demonstrated acceptable test–retest reliability and showed a positive correlation with the latent classroom peer relations construct, consistent with theoretical expectations. These findings provide support for its reliability and convergent validity, in line with recommended approaches for single-item measures (Allen et al. 2022).<br><br>However, we have avoided overinterpretation of findings related to classroom climate and revised the Limitations to better	may be more susceptible to measurement error than multi-item scales, thereby limiting the precision of estimated associations. As a result, associations involving classroom climate should be interpreted with caution. Future studies would benefit from using more comprehensive measures to capture classroom climate in greater detail."

	clarify the conceptual limitations of the measure.	
10. Minor comments:<br><br>The Introduction is good, but it could do a better job distinguishing peer ecology as a broad social context, PA-specific peer support, and classroom climate.	We have revised the Introduction to more clearly distinguish between classroom peer ecology, classroom climate, and PA-specific peer support, based on this helpful suggestion.	**Introduction**, line numbers 103–113:<br><br>"Despite a growing body of research on student peer outcomes and PA, much of it focuses on overall PA [29,32] or PA-specific peer support, which captures more narrowly defined behaviors directly related to PA (e.g., encouragement, shared activities, team sports participation) [17-19,33]. In contrast, research on how broader classroom social contexts are associated with PA and ST during school hours is still in its infancy [3,34], and even less is known about potential gender differences in these relationships. The current study focuses on students' individual perceptions of the classroom peer ecology as a broader social context, including peer relations in the classroom and classroom climate. Classroom climate reflects overall perceptions of the classroom environment, whereas peer relations refer more specifically to the quality of relationships among classmates, including acceptance, friendliness, and support. Accordingly, we examine how the perceived classroom peer ecology relates to students' in-school PA and ST and academic achievement."
11. The limitations section is one of the stronger parts of the manuscript. In fact, it is more cautious than the discussion and conclusion. The Reviewer recommends using the	We agree that maintaining a consistent and appropriately cautious tone throughout the manuscript is important for	Please see the changes made in #6, #7, #8 and #9.

limitations section as the tone standard for the whole paper.	accurately reflecting the observational nature of the study.<br><br>We believe the revisions in response to the Reviewer's previous comments and suggestions have improved consistency in tone across the manuscript, and that it now better matches the tone of the Limitations section.	
12. Students do not experience activity opportunities only as physical opportunities; they experience them through belonging, peer norms, identity, and classroom culture. The Reviewers ask the Authors to consider making their practice message more about designing activity opportunities that are socially inclusive, not assuming all students experience active school practices in the same way, and considering how classroom and recess social norms shape participation.	We fully agree with this well-articulated perspective. We have revised the more practical messages in the Abstract, Discussion, and Conclusions to better include this viewpoint.	**Abstract,** line numbers 46–48:<br><br>"Efforts to promote a physically active school day could benefit from considering how activity opportunities are experienced within peer dynamics and in relation to gender, including the need for socially inclusive approaches."<br><br>**Discussion**, line numbers 369–373:<br><br>"From a practical perspective, efforts to promote PA in schools should not focus solely on increasing opportunities, but also on ensuring that these opportunities are socially inclusive. Educators should consider how classroom and recess norms, peer dynamics, and students' sense of belonging may facilitate or hinder participation."<br><br>**Conclusions**, line numbers 447–449:<br><br>"Practically, the results suggest the potential value of considering how students experience in-school PA opportunities within their social environment when designing PA provision during the school day and pedagogical practices,

		although causal interpretations cannot be made.”

References cited in the response to reviewers:

Allen, M. S., Iliescu, D., & Greiff, S. (2022). Single item measures in psychological science: a call to action. European Journal of Psychological Assessment, 38(1), 1–5. doi: 10.1027/1015-5759/a000699.

Haapala, H., Hirvensalo, M., Laine, K., Laakso, L., Hakonen, H., Kankaanpää, A., et al. Recess physical activity and school-related social factors in Finnish primary and lower secondary schools: cross-sectional associations. BMC Public Health. 2014;14(1):1114. doi:10.1186/1471-2458-14-1114.

Song, J., Howe, E., Oltmanns, J., & Fisher, A. (2023). Examining the concurrent and predictive validity of single items in ecological momentary assessments. Assessment, 30(5), 1662–1671. doi: 10.1177/10731911221113563.

References added to the manuscript:

Andersen, H. B., Christiansen, L. B., Pawlowski, C. S., & Schipperijn, J. (2019). What we build makes a difference – Mapping activating schoolyard features after renewal using GIS, GPS and accelerometers. Landscape and Urban Planning, 191, 103617. doi: 10.1016/j.landurbplan.2019.103617.
