# Supplementary material for: Gendered patterns in the associations between in-school physical activity, classroom peer ecology, and academic achievement among primary school students
Source: PLoS One. 2026 Jun 22;21(6):e0351971. doi: 10.1371/journal.pone.0351971 (PMC13286205; doi:10.1371/journal.pone.0351971)
Supplement: Attachment — Submitted filename: Response_to_Reviewers_auresp_3.pdf [file pone.0351971.s004.pdf]

POINT-BY-POINT RESPONSE TO REVIEWERS

Ms. Ref. No.: PONE-D-25-55757R3
Manuscript title: Gendered patterns in the associations between in-school physical activity, classroom peer ecology, and academic achievement among primary school students

Note: All line numbers referred to in this response correspond to the revised, unmarked version of the manuscript (labeled ‘Manuscript’).

**Suggestion, question, or comment from Reviewer #4**	Authors’ response	Change in the manuscript
1. The Reviewer thanks the Authors for responding thoughtfully to the prior comment. A couple of things to address briefly:<br><br>Was the classroom climate item reverse-coded? Please clarify.	We thank the reviewer for these helpful additional comments and questions.<br><br>The labels in parentheses had previously been incorrectly reversed and have now been corrected.	**Methods,** line numbers 163–164:<br><br>“Students responded to the questions on a five-point scale ranging from 1 (very poor) to 5 (very good).”
2. The Reviewer believes that altering the title to "Gendered patterns in the associations..." is more fitting and would match the conclusion by the Authors better (i.e., patterns were different, but not always statistically different between gender)	We thank the reviewer for this suggestion. We agree that the suggested version better reflects the manuscript. Accordingly, the title has now been revised.	**Title**, line number 4–6:<br><br>“Gendered patterns in the associations between in-school physical activity, classroom peer ecology, and academic achievement among primary school students”
3. For Table 4, just make sure the direction of Cohen's d are all correct	We double-checked the calculations for Cohen’s d for all variables in Table 4. We now confirm that the directions of Cohen’s d values are consistent with the group coding (girls vs. boys) and the mean differences.	**Results**, Table 4:<br><br>The sign for in-school MVPA has been corrected to negative, reflecting lower in-school MVPA among girls compared to boys.<br><br>Line number 268:<br>“Note: [a] positive values indicate higher scores among girls”<br><br>In addition, some minor changes were made to the values in Table 4 after rerunning the Cohen’s d calculations.

4. For Tables 5 and 6, upper should probably be 97.5% rather than 2.5%. Conventionally, the Reviewer recommends 95% CI lower and upper.	The original labels followed the terminology used in the Mplus output, but we agree with the reviewer that the clarity could be improved. Accordingly, we revised the tables 5 and 6.	**Results**, Table 5 and 6:<br><br>We revised the tables to present the confidence intervals in a single column labeled "95% CI".
5. From the Reviewer's scientific education and bias, a p-value should never be 0.000 - please report at $p < 0.001$	We thank the reviewer for pointing this out. Tables 4, 5, and 6 have now been revised.	**Results**, Tables 4–6:<br><br>Reported at $p<.001$ where applicable.
**Suggestion, question, or comment from the Academic Editor**	Authors' response	Change in the manuscript
6. The revised manuscript has improved substantially and addresses the majority of the reviewers' and editor's previous concerns. The study addresses an important and timely topic, and the analyses are generally appropriate and carefully conducted. The manuscript is now close to being suitable for publication. Before a final decision can be made, however, the authors are requested to address several remaining issues through a minor revision:<br><br>Interpretation of gender differences: While the manuscript appropriately reports some observed differences in association patterns between girls and boys, several formal between-group comparisons were not statistically significant. Please further ensure throughout the Abstract, Results, Discussion, and Conclusions that interpretations remain cautious and do not overstate gender-specific effects where statistical evidence for group differences is limited.	We sincerely thank the editor for taking the time for the insightful evaluation of our manuscript.<br><br>We have carefully revised the manuscript throughout to ensure that interpretations of gendered patterns are more cautious and consistent with the statistical findings. We note that the observed subgroup patterns are based on differences in parameter estimates within the multigroup SEM framework, but we have ensured that these are not overinterpreted in the absence of statistically significant between-group differences. Accordingly, we have avoided wording implying confirmed gender-specific effects and frame these findings as suggestive patterns requiring cautious interpretation.<br><br>In addition, in line with the 2nd suggestion from the Reviewer #4, we have revised the title to better fit the manuscript.	**Abstract,** line numbers 42–47:<br><br>"Although the MVPA-related associations differed in statistical significance across groups, subsequent formal tests did not indicate statistically significant gender differences, and hence further studies are needed on the subgroup-specific patterns to confirm or invalidate the findings. Efforts to promote a physically active school day could benefit from considering how activity opportunities are experienced within peer dynamics and in relation to gender, including the need for socially inclusive approaches."<br><br>**Discussion,** line numbers 313–314:<br><br>"The findings identified some gendered patterns in the dynamics between the perceived peer ecology, in-school MVPA, in-school ST, and academic achievement."<br><br>Line numbers 315–327:

		"In the current study, more positive perceptions of classroom peer relations were significantly associated with less time spent in in-school MVPA among girls, whereas this association was not statistically significant among boys. Although the association observed differed in statistical significance across groups, subsequent tests of gender differences were not statistically significant, and thus the findings concerning gendered pattern should be interpreted cautiously. Previous research shows that social dynamics can shape students' engagement in in-school PA [10], and the relationship between social dynamics and activity behaviors may vary by gender [29-31]. While prior studies have suggested that PA can support social competence and peer relationships [12-13] and that peer-related social factors are positively associated with higher PA levels [14-15,41], the present study did not find evidence linking more positive classroom peer dynamics with in-school MVPA.<br><br>This unexpected finding may suggest a potential inverse association between self-reported peer relations in the classroom and engagement in more intense forms of in-school PA, particularly in the descriptive pattern observed among girls."<br><br>Line numbers 367–372:

		"While participation in physical activities provides opportunities for peer interaction [67], our results suggest that more positive self-reported peer relations in the classroom do not necessarily correspond to higher levels of in-school MVPA. Taken together, these findings suggest differences in the magnitude of the association between social experiences and in-school PA between girls and boys, although these should be interpreted cautiously given the lack of statistically significant gender differences."<br><br>Line numbers 392–395:<br><br>"The results further suggested a positive association between MVPA and GPA among boys, whereas no significant association was observed among girls. However, this pattern did not translate into a statistically significant between-group difference and should be interpreted as a descriptive pattern."<br><br>Line numbers 401–404:<br><br>"Against this background, the present study focused on objectively measured in-school MVPA, and the positive association observed between in-school MVPA and GPA, at least among boys, is consistent with the overall direction of effects reported by He et al."<br><br>**Limitations**, line numbers 444–445:<br><br>"The gender differences in the associations involving in-school

		MVPA warrant cautious interpretation, since formal gender comparisons did not reveal statistically significant differences."<br><br>**Conclusions,** line numbers 453–461:<br><br>"This study examined the dynamics between in-school PA and ST, classroom peer ecology, and academic achievement. In girls, more positive self-reported classroom peer relations were associated with lower levels of in-school MVPA, while greater in-school ST was linked to higher GPA. Among boys, higher in-school MVPA was related to higher GPA. Although the MVPA-related associations differed in statistical significance across groups, subsequent formal tests did not indicate statistically significant gender differences, and these subgroup-specific patterns should therefore be interpreted cautiously. Practically, the results suggest the potential value of considering how students experience in-school PA opportunities within their social environment when designing PA provision during the school day and pedagogical practices, although causal interpretations cannot be made."
7. Data availability statement: Please revise the Data Availability Statement to ensure full compliance with PLOS ONE data-sharing requirements. In particular, the justification for restrictions on data access should be clarified further, including a more explicit explanation of the ethical or legal constraints	Accordingly, we have further revised the Data Availability Statement:<br><br>"Data for this study were collected as part of a larger research project called Students' Physical Activity and Well-being. All the data are owned by Likes, Jamk University of	

preventing public deposition of the dataset and the exact conditions under which data access may be granted.	Applied Sciences. Restrictions apply to the availability of these data as the ethical approval received at the time of the data collection did not permit open public sharing of the dataset. The data were used under a license agreement for the current study and are therefore not publicly available. Researchers seeking access should submit a reasonable request including a research plan to the Ethics Committee of Jamk University of Applied Sciences (contact: eettinentk@jamk.fi), after which data access will be evaluated on a case-by-case basis. Access to the data is subject to a separate data use agreement that specifies the terms and conditions for data transfer, storage, and use."	
8. Classroom climate measure: The use of a single-item measure for classroom climate is acknowledged as a limitation. Please strengthen the methodological justification for this choice in the Methods section and briefly elaborate on the implications this may have for interpretation of the findings.	We have now strengthened the methodological justification for the classroom climate measure in the Methods section by clarifying its role, practicality, reliability, and observed convergent validity. In addition, we have expanded the Limitations section to briefly clarify the implications of this measurement approach for the interpretation of findings.	**Methods,** line numbers 164–171:<br><br>"The item was included to capture students' general perceptions of the classroom environment and to complement the more detailed measure of classroom peer relations. Brief single-item measures may also reduce participant burden and can provide a straightforward indicator with acceptable face validity in large school-based surveys. Although single-item measures cannot fully capture the multidimensional nature of classroom climate, similar brief indicators have been used in prior research [41]. The item demonstrated good test–retest reliability (ICC = 0.73, 95% CI: 0.66–0.79) and showed a positive correlation with the latent classroom peer relations construct, consistent with theoretical expectations."

		**Limitations**, line numbers 421–427:<br><br>"In the present study, classroom climate was assessed using a single-item measure, which cannot fully capture the complexity of the construct and may be more susceptible to measurement error than multi-item scales, thereby limiting the precision of estimated associations. Consequently, associations involving classroom climate should be interpreted cautiously, as the measure reflects a broad general perception of the classroom climate rather than specific dimensions of the construct. Future studies would benefit from using more comprehensive measures to capture classroom climate in greater detail."
9. Relevance of older data collection period: As the data were collected in 2013, please further clarify in the Discussion why the findings remain relevant to contemporary school contexts and current educational or physical activity practices.	We have revised the manuscript to clarify the contemporary relevance of the data by adding a concise explanation in the Discussion section. We also streamlined the corresponding part in the Limitations section to avoid redundancy while still acknowledging temporal and contextual considerations for generalizability.	**Discussion,** line numbers 405–412:<br><br>"Although the data were collected in 2013, the findings remain relevant for contemporary school contexts. The present study focused on associations between in-school PA and ST, classroom peer ecology, and academic achievement, rather than on characterizing current patterns of students' behaviors or experiences. While educational practices and in-school PA promotion have continued to evolve, the core structures of Finnish primary education, such as classroom-based instruction and stable peer groups, as well as the importance of peer

		interactions in students' daily school experiences, largely remain unchanged. Thus, the associations may still provide meaningful insights for current efforts to promote physically active and socially inclusive school environments."<br><br>**Limitations**, line numbers 415–420:<br><br>"The data were collected in 2013, and while educational practices and technological influences have continued to evolve since then, the key constructs examined remain relevant in Finnish primary education, and the relationships are unlikely to depend on a particular curriculum version. Nevertheless, changes in curriculum frameworks and broader behavioral contexts should be considered when generalizing the findings to current cohorts or to other educational systems."
10. Please carefully proofread the manuscript once more for clarity, consistency of terminology, and minor grammatical or stylistic issues.	The manuscript has now been carefully proofread to improve clarity, consistency of terminology, and to correct minor grammatical and stylistic issues. In addition, the revised manuscript was reviewed by a professional language editor.	**Throughout the manuscript:**<br><br>Where applicable, terminology was standardized throughout the manuscript, including consistent use of "academic achievement" and "in-school PA" and "in-school ST" (previously also referred to as, e.g., "academic outcomes," "academic performance," "PA during school days," and "ST during school days,")<br><br>In-text citations were corrected and standardized to ensure consistent use of numerical citation order formatting. Instances of mixed citation styles and unnecessary

		inclusion of publication years in the text were removed<br><br>Minor grammatical corrections were made (e.g., subject–verb agreement and article usage)<br><br>Punctuation was corrected and standardized throughout the manuscript<br><br>Typographical issues, including removal of double spaces, were corrected<br><br>Readability was improved through minor sentence-level revisions<br><br>**Results**, Tables 4-6:<br><br>Table 4 was updated, including bold formatting of titles in the first column<br><br>Tables 5 and 6 were revised in accordance with Reviewer #4's comment no. 4.
