# Supplementary material for: Gendered patterns in the associations between in-school physical activity, classroom peer ecology, and academic achievement among primary school students
Source: PLoS One. 2026 Jun 22;21(6):e0351971. doi: 10.1371/journal.pone.0351971 (PMC13286205; doi:10.1371/journal.pone.0351971)
Supplement: Peer review history

# Peer review: Decision Letter 0

**DOI:** 10.1371/journal.pone.0351971.r001

**Authors:** Georgian Badicu

31 Dec 2025

PONE-D-25-55757Gender-specific dynamics between in-school physical activity, classroom peer ecology, and academic achievement among primary school studentsPLOS One

Dear Dr.  Byman,

Thank you for submitting your manuscript to PLOS ONE. After careful consideration, we feel that it has merit but does not fully meet PLOS ONE’s publication criteria as it currently stands. Therefore, we invite you to submit a revised version of the manuscript that addresses the points raised during the review process.

Please submit your revised manuscript by Feb 14 2026 11:59PM. If you will need more time than this to complete your revisions, please reply to this message or contact the journal office at plosone@plos.org. When you're ready to submit your revision, log on to https://www.editorialmanager.com/pone/ and select the 'Submissions Needing Revision' folder to locate your manuscript file.

Please include the following items when submitting your revised manuscript:

- A letter that responds to each point raised by the academic editor and reviewer(s). You should upload this letter as a separate file labeled 'Response to Reviewers'.
- A marked-up copy of your manuscript that highlights changes made to the original version. You should upload this as a separate file labeled 'Revised Manuscript with Track Changes'.
- An unmarked version of your revised paper without tracked changes. You should upload this as a separate file labeled 'Manuscript'.

If you would like to make changes to your financial disclosure, please include your updated statement in your cover letter. Guidelines for resubmitting your figure files are available below the reviewer comments at the end of this letter.

If applicable, we recommend that you deposit your laboratory protocols in protocols.io to enhance the reproducibility of your results. Protocols.io assigns your protocol its own identifier (DOI) so that it can be cited independently in the future. For instructions see: https://journals.plos.org/plosone/s/submission-guidelines#loc-laboratory-protocols. Additionally, PLOS ONE offers an option for publishing peer-reviewed Lab Protocol articles, which describe protocols hosted on protocols.io. Read more information on sharing protocols at https://plos.org/protocols?utm_medium=editorial-email&utm_source=authorletters&utm_campaign=protocols.

We look forward to receiving your revised manuscript.

Kind regards,

Georgian Badicu, Ph.D

Academic Editor

PLOS One

Journal Requirements:

When submitting your revision, we need you to address these additional requirements.

1.Please ensure that your manuscript meets PLOS ONE's style requirements, including those for file naming. The PLOS ONE style templates can be found at

https://journals.plos.org/plosone/s/file?id=wjVg/PLOSOne_formatting_sample_main_body.pdf and

https://journals.plos.org/plosone/s/file?id=ba62/PLOSOne_formatting_sample_title_authors_affiliations.pdf

2. Thank you for stating the following financial disclosure:

“Data collection for this study was supported by a grant (OKM/92/626/2013) from the Finnish Ministry of Education and Culture and the Juho Vainio Foundation. This manuscript was prepared as part of the SchoolWell project, which was funded by the Strategic Research Council established within the Research Council of Finland [353361 (T.H.T.) and 1252509 (K.P)].”

Please state what role the funders took in the study.  If the funders had no role, please state: "The funders had no role in study design, data collection and analysis, decision to publish, or preparation of the manuscript."

If this statement is not correct you must amend it as needed.

Please include this amended Role of Funder statement in your cover letter; we will change the online submission form on your behalf.

3. In the online submission form you indicate that your data is not available for proprietary reasons and have provided a contact point for accessing this data. Please note that your current contact point is a co-author on this manuscript. According to our Data Policy, the contact point must not be an author on the manuscript and must be an institutional contact, ideally not an individual. Please revise your data statement to a non-author institutional point of contact, such as a data access or ethics committee, and send this to us via return email. Please also include contact information for the third party organization, and please include the full citation of where the data can be found.

4. Please include your full ethics statement in the ‘Methods’ section of your manuscript file. In your statement, please include the full name of the IRB or ethics committee who approved or waived your study, as well as whether or not you obtained informed written or verbal consent. If consent was waived for your study, please include this information in your statement as well.

5. If the reviewer comments include a recommendation to cite specific previously published works, please review and evaluate these publications to determine whether they are relevant and should be cited. There is no requirement to cite these works unless the editor has indicated otherwise.

Additional Editor Comments:

Major and substantive revisions are required for this manuscript. The reviewers’ comments are pertinent, and the authors should address each comment thoroughly and appropriately.

[Note: HTML markup is below. Please do not edit.]

Reviewers' comments:

Reviewer's Responses to Questions

**Comments to the Author**

1. Is the manuscript technically sound, and do the data support the conclusions?

The manuscript must describe a technically sound piece of scientific research with data that supports the conclusions. Experiments must have been conducted rigorously, with appropriate controls, replication, and sample sizes. The conclusions must be drawn appropriately based on the data presented.

Reviewer #1: Yes

Reviewer #2: Yes

Reviewer #3: No

**********

2. Has the statistical analysis been performed appropriately and rigorously?

Reviewer #1: Yes

Reviewer #2: Yes

Reviewer #3: No

**********

3. Have the authors made all data underlying the findings in their manuscript fully available?

The PLOS Data policy requires authors to make all data underlying the findings described in their manuscript fully available without restriction, with rare exception (please refer to the Data Availability Statement in the manuscript PDF file). The data should be provided as part of the manuscript or its supporting information, or deposited to a public repository. For example, in addition to summary statistics, the data points behind means, medians and variance measures should be available. If there are restrictions on publicly sharing data—e.g. participant privacy or use of data from a third party—those must be specified.

Reviewer #1: Yes

Reviewer #2: Yes

Reviewer #3: No

**********

4. Is the manuscript presented in an intelligible fashion and written in standard English?

PLOS ONE does not copyedit accepted manuscripts, so the language in submitted articles must be clear, correct, and unambiguous. Any typographical or grammatical errors should be corrected at revision, so please note any specific errors here.

Reviewer #1: Yes

Reviewer #2: Yes

Reviewer #3: Yes

**********

5. Review Comments to the Author

Please use the space provided to explain your answers to the questions above. You may also include additional comments for the author, including concerns about dual publication, research ethics, or publication ethics. (Please upload your review as an attachment if it exceeds 20,000 characters)

Reviewer #1: Interesting idea of this study, my recommendations are the following:

Abstract – I recommend rewriting the first sentence, mention ... for increasing physical activity (PA), it is not clear and logical, you can possibly use another verb such as: practicing or increasing the duration of practicing PA or actively involving in PA.

I recommend reviewing the keywords, certain words do not appear from the title of the study such as: Sport and exercise sciences; Social sciences. Also Academic achievement; Academic performance; in conclusion, I recommend clarifications.

Line 20- PA cannot increase, this association of words is inappropriate, I recommend rewriting.

Primary education in Finland – I recommend mentioning how many hours/week of PA are provided for in the curriculum in primary education.

Participants and procedures – according to the information regarding the year of this study, I recommend adding clarifications regarding the difference of 12 years that have passed since 2013. I also recommend mentioning whether in those 12 years there have been changes to the curriculum structure at the primary education level. In my opinion, 12 years represents a major gap both socially and psychologically due to technological and methodological influences at the educational level. I recommend making justifications regarding the aforementioned.

I recommend that all acronyms be mentioned descriptively under the tables.

In conclusion, the study is well organized, the information has a scientific logic and is relevant.

Reviewer #2: The purpose of this study was to examine how classroom peer ecology is associated with PA and ST during school hours, and how these factors relate to academic achievement among primary school students. Subjects were 517 Finnish primary school students (53% girls, 47% boys, Mage = 11.6 years) participated in this study in 2013. Students’ perceptions of classroom peer ecology were investigated through a survey. In addition, moderate-to-vigorous PA (MVPA) and ST during school hours were measured by hip-worn accelerometers. Moreover, grade point averages were calculated based on subject grades. Besides, gender-specific associations were investigated through multigroup structural equation modeling analyses. Results of this study indicated that, positive classroom peer relations were associated with lower levels of in-school MVPA, while greater in-school ST was linked to higher grade point average in girls. However, among boys, higher in-school MVPA was related to higher grade point average. No significant associations were found between social variables and in-school ST.

I would like to thank to authors for this interesting study. I believe authors made a such nice introduction section with hypothesis and their problem of statement is vey clear. In addition, this study had a good sample size and appropriate measurement. My question is about validity and reliability of tests and measurements. This should be reported in the study. Moreover, this study needs a strong discussion and conclusion sections with up to date literature review (2023 and above) with practical suggestions for the future. I look forward to seeing edited version of this timely and interesting manuscript. Thank you.

Reviewer #3: Dear Authors,

Thank you for submitting your manuscript. The topic is potentially relevant; however, in its current form the study has several fundamental issues that substantially limit the credibility, reproducibility, and interpretability of the findings. For these reasons, I recommend rejection.

Data availability and compliance with PLOS Data Policy

PLOS requires that all data underlying the reported findings be made fully available without restriction. In the current submission, the underlying dataset is not clearly provided in an unrestricted, accessible form, and the description suggests that access may depend on requesting data from the authors/third parties. This does not meet the expected standard for transparency and reproducibility unless a clearly justified exception is documented and an alternative compliant solution is provided.

Major statistical validity concern: clustered (hierarchical) data not appropriately modeled

The data structure is clustered (e.g., students nested within classes/schools). While the manuscript acknowledges intraclass correlation (ICC), it also states that the final modeling did not incorporate clustering due to technical limitations. This is a serious methodological flaw because ignoring the hierarchical structure can bias standard errors and p-values, inflating statistical significance and undermining inferential validity. This issue affects the core conclusions and cannot be treated as a minor limitation.

Limited explanatory value and weak practical significance

The reported models appear to have very low explanatory power (small R² values) with only a small number of statistically significant pathways. As a result, the added scientific value is limited and the manuscript risks overemphasizing marginal effects that may not be robust.

Study design constraints and temporal relevance

The cross-sectional design does not support causal inference, yet parts of the discussion and implications drift toward causal language and strong recommendations. In addition, the dataset is relatively old, and the manuscript does not sufficiently justify the contemporary relevance and generalizability of the findings to current educational/behavioral contexts.

Over-interpretation of non-intuitive associations without adequate robustness checks

Some reported associations are not intuitive (e.g., unexpected directions of relationships) and are explained largely through post-hoc speculation. The manuscript would require stronger triangulation, sensitivity analyses, and/or alternative model specifications to support such interpretations.

Measurement limitations for key constructs

Using very brief or single-item measures for important constructs (e.g., classroom climate) raises concerns about measurement error and construct validity especially when these measures are used in complex models (e.g., SEM). This further weakens confidence in the reported pathways.

Given the combination of (i) data availability concerns, and (ii) a critical modeling limitation related to clustered data alongside low explanatory power and interpretive overreach I do not believe the manuscript is suitable for publication in its current form.

**********

6. PLOS authors have the option to publish the peer review history of their article (what does this mean?). If published, this will include your full peer review and any attached files.

If you choose “no”, your identity will remain anonymous but your review may still be made public.

**Do you want your identity to be public for this peer review?**  For information about this choice, including consent withdrawal, please see our Privacy Policy.

Reviewer #1: **Yes:**  Badau Adela

Reviewer #2: **Yes:**  Ferman Konukman

Reviewer #3: No

**********

[NOTE: If reviewer comments were submitted as an attachment file, they will be attached to this email and accessible via the submission site. Please log into your account, locate the manuscript record, and check for the action link "View Attachments". If this link does not appear, there are no attachment files.]

To ensure your figures meet our technical requirements, please review our figure guidelines: https://journals.plos.org/plosone/s/figures

You may also use PLOS’s free figure tool, NAAS, to help you prepare publication quality figures: https://journals.plos.org/plosone/s/figures#loc-tools-for-figure-preparation.

NAAS will assess whether your figures meet our technical requirements by comparing each figure against our figure specifications.

# Author comment: Author response to Decision Letter 1

**DOI:** 10.1371/journal.pone.0351971.r002

17 Feb 2026

We sincerely thank the reviewers for their constructive and helpful comments. Please see the uploaded document titled “Response to Reviewers” for a detailed, point-by-point response. All corresponding revisions have been incorporated into the manuscript.

Attachment Submitted filename: Response to Reviewers.pdf

# Peer review: Decision Letter 1

**DOI:** 10.1371/journal.pone.0351971.r003

**Authors:** Georgian Badicu, Georgian Badicu

30 Mar 2026

PONE-D-25-55757R1Gender-specific dynamics between in-school physical activity, classroom peer ecology, and academic achievement among primary school studentsPLOS One

Dear Dr. Byman,

Thank you for submitting your manuscript to PLOS ONE. After careful consideration, we feel that it has merit but does not fully meet PLOS ONE’s publication criteria as it currently stands. Therefore, we invite you to submit a revised version of the manuscript that addresses the points raised during the review process.

Please submit your revised manuscript by May 14 2026 11:59PM. If you will need more time than this to complete your revisions, please reply to this message or contact the journal office at plosone@plos.org. When you're ready to submit your revision, log on to https://www.editorialmanager.com/pone/ and select the 'Submissions Needing Revision' folder to locate your manuscript file.

Please include the following items when submitting your revised manuscript:

- A letter that responds to each point raised by the academic editor and reviewer(s). You should upload this letter as a separate file labeled 'Response to Reviewers'.
- A marked-up copy of your manuscript that highlights changes made to the original version. You should upload this as a separate file labeled 'Revised Manuscript with Track Changes'.
- An unmarked version of your revised paper without tracked changes. You should upload this as a separate file labeled 'Manuscript'.

If you would like to make changes to your financial disclosure, please include your updated statement in your cover letter. Guidelines for resubmitting your figure files are available below the reviewer comments at the end of this letter.

If applicable, we recommend that you deposit your laboratory protocols in protocols.io to enhance the reproducibility of your results. Protocols.io assigns your protocol its own identifier (DOI) so that it can be cited independently in the future. For instructions see: https://journals.plos.org/plosone/s/submission-guidelines#loc-laboratory-protocols. Additionally, PLOS ONE offers an option for publishing peer-reviewed Lab Protocol articles, which describe protocols hosted on protocols.io. Read more information on sharing protocols at https://plos.org/protocols?utm_medium=editorial-email&utm_source=authorletters&utm_campaign=protocols.

As the corresponding author, your ORCID iD is verified in the submission system and will appear in the published article. PLOS supports the use of ORCID, and we encourage all coauthors to register for an ORCID iD and use it as well. Please encourage your coauthors to verify their ORCID iD within the submission system before final acceptance, as unverified ORCID iDs will not appear in the published article. *Only*  the individual author can complete the verification step; PLOS staff *cannot*  verify ORCID iDs on behalf of authors.

We look forward to receiving your revised manuscript.

Kind regards,

Georgian Badicu, Ph.D

Academic Editor

PLOS One

Journal Requirements:

If the reviewer comments include a recommendation to cite specific previously published works, please review and evaluate these publications to determine whether they are relevant and should be cited. There is no requirement to cite these works unless the editor has indicated otherwise.

Additional Editor Comments:

Please ensure that all reviewers’ comments are addressed clearly and comprehensively, where appropriate.

Kind regards!

[Note: HTML markup is below. Please do not edit.]

Reviewers' comments:

Reviewer's Responses to Questions

**Comments to the Author**

1. If the authors have adequately addressed your comments raised in a previous round of review and you feel that this manuscript is now acceptable for publication, you may indicate that here to bypass the “Comments to the Author” section, enter your conflict of interest statement in the “Confidential to Editor” section, and submit your "Accept" recommendation.

Reviewer #1: All comments have been addressed

Reviewer #2: All comments have been addressed

Reviewer #4: (No Response)

**********

2. Is the manuscript technically sound, and do the data support the conclusions?

The manuscript must describe a technically sound piece of scientific research with data that supports the conclusions. Experiments must have been conducted rigorously, with appropriate controls, replication, and sample sizes. The conclusions must be drawn appropriately based on the data presented.

Reviewer #1: Yes

Reviewer #2: Yes

Reviewer #4: Partly

**********

3. Has the statistical analysis been performed appropriately and rigorously?

Reviewer #1: Yes

Reviewer #2: Yes

Reviewer #4: Yes

**********

4. Have the authors made all data underlying the findings in their manuscript fully available?

The PLOS Data policy requires authors to make all data underlying the findings described in their manuscript fully available without restriction, with rare exception (please refer to the Data Availability Statement in the manuscript PDF file). The data should be provided as part of the manuscript or its supporting information, or deposited to a public repository. For example, in addition to summary statistics, the data points behind means, medians and variance measures should be available. If there are restrictions on publicly sharing data—e.g. participant privacy or use of data from a third party—those must be specified.

Reviewer #1: Yes

Reviewer #2: Yes

Reviewer #4: Yes

**********

5. Is the manuscript presented in an intelligible fashion and written in standard English?

PLOS ONE does not copyedit accepted manuscripts, so the language in submitted articles must be clear, correct, and unambiguous. Any typographical or grammatical errors should be corrected at revision, so please note any specific errors here.

Reviewer #1: Yes

Reviewer #2: Yes

Reviewer #4: Yes

**********

6. Review Comments to the Author

Please use the space provided to explain your answers to the questions above. You may also include additional comments for the author, including concerns about dual publication, research ethics, or publication ethics. (Please upload your review as an attachment if it exceeds 20,000 characters)

Reviewer #1: No comments. The authors have revised the article in accordance with the above recommendations. I believe that in its current form the article is ready for the journal.

Reviewer #2: I believe the current edited version this manuscript is acceptable. All my questions answered in detail. Thank you.

Reviewer #4: This is an interesting manuscript with a meaningful contribution using objectively measured in-school movement behaviours and academic achievement in primary school students. That combination is valuable, especially because the study uses accelerometer-based school-time MVPA/ST rather than relying entirely on self-report, and because it takes the school/classroom context seriously rather than treating activity as a purely individual behaviour. The research questions are clear and the manuscript is generally well organized. Furthermore, it is very sound science using objectively measured in-school MVPA and ST, the methods are clear and the modeling is well thought out. Most importantly, it has a practical resonance for educators.

Major concerns:

The manuscript repeatedly emphasizes "gendered" or "gender-specific" patterns in the abstract, discussion, and conclusion. But in the MVPA model, the authors explicitly report that constraining the significant paths to equality across genders did not significantly worsen fit, meaning those associations did not significantly differ by gender. Yes, the authors did disclose in the limitations, but the big picture is that the paper cannot say that the MVPA associations did not significantly differ by gender! Interpretation of gender has to be consistent across Results, Discussion, Conclusion...

The study is cross-sectional, the data were collected in 2013, and the explained variance in GPA is quite modest. The models explain only a small proportion of GPA variance, and the authors themselves note that the findings should be interpreted as individual-level associations, not classroom-level effects. However, the manuscript sometimes sounds close to recommending how schools should design physically active school days based on these findings. The Reviewer believes the evidence base here is not strong enough for broad pedagogical recommendations.

The negative association between peer relations and girls' in-school MVPA is interesting, but much of the explanation offered in the discussion goes beyond what the data can support. The manuscript suggests girls may avoid vigorous activity due to social discomfort or exclusion, or that peer norms may favour lower-intensity activity. These are plausible ideas, but they were not measured directly. Consider offering 2-3 alternative explanations with equal weight, including measurement limitations of hip-worn accelerometers and the possibility that socially valued activity for girls may not register well as MVPA.

"Classroom climate" is a rich construct that usually involves emotional safety, norms, teacher support, belonging, participation structure, and sometimes behaviour management or instructional organization. A single item on how students experience the class climate is unlikely to capture that complexity. Explain why this still adds value despite being conceptually thin and avoid overinterpreting null findings for classroom climate.

Minor comments:

The Introduction is good, but it could do a better job distinguishing peer ecology as a broad social context, PA-specific peer support, and classroom climate.

The limitations section is one of the stronger parts of the manuscript. In fact, it is more cautious than the discussion and conclusion. The Reviewer recommends using the limitations section as the tone standard for the whole paper.

Students do not experience activity opportunities only as physical opportunities; they experience them through belonging, peer norms, identity, and classroom culture. The Reviewers ask the Authors to consider making their practice message more about designing activity opportunities that are socially inclusive, not assuming all students experience active school practices in the same way, and considering how classroom and recess social norms shape participation.

**********

7. PLOS authors have the option to publish the peer review history of their article (what does this mean?). If published, this will include your full peer review and any attached files.

If you choose “no”, your identity will remain anonymous but your review may still be made public.

**Do you want your identity to be public for this peer review?**  For information about this choice, including consent withdrawal, please see our Privacy Policy.

Reviewer #1: **Yes:**  Adela Badau

Reviewer #2: **Yes:**  Ferman Konukman

Reviewer #4: **Yes:**  Shane J.T. Balthazaar

[NOTE: If reviewer comments were submitted as an attachment file, they will be attached to this email and accessible via the submission site. Please log into your account, locate the manuscript record, and check for the action link "View Attachments". If this link does not appear, there are no attachment files.]

To ensure your figures meet our technical requirements, please review our figure guidelines: https://journals.plos.org/plosone/s/figures

You may also use PLOS’s free figure tool, NAAS, to help you prepare publication quality figures: https://journals.plos.org/plosone/s/figures#loc-tools-for-figure-preparation.

NAAS will assess whether your figures meet our technical requirements by comparing each figure against our figure specifications.

# Author comment: Author response to Decision Letter 2

**DOI:** 10.1371/journal.pone.0351971.r004

12 May 2026

We sincerly thank the reviewers for the careful evaluation of our manuscript and for the constructive comments provided. We have carefully considered all comments and have revised the manuscript accordingly. A detailed, point-by-point response is provided in a separate document, and all corresponding changes have been indicated in the revised manuscript.

Attachment Submitted filename: Response_to_Reviewers_auresp_2.pdf

# Peer review: Decision Letter 2

**DOI:** 10.1371/journal.pone.0351971.r005

**Authors:** Georgian Badicu, Georgian Badicu, Georgian Badicu

18 May 2026

PONE-D-25-55757R2Gender-specific dynamics between in-school physical activity, classroom peer ecology, and academic achievement among primary school studentsPLOS One

Dear Dr. Byman,

Thank you for submitting your manuscript to PLOS ONE. After careful consideration, we feel that it has merit but does not fully meet PLOS ONE’s publication criteria as it currently stands. Therefore, we invite you to submit a revised version of the manuscript that addresses the points raised during the review process.

==============================

**ACADEMIC EDITOR:**

The revised manuscript has improved substantially and addresses the majority of the reviewers’ and editor’s previous concerns. The study addresses an important and timely topic, and the analyses are generally appropriate and carefully conducted. The manuscript is now close to being suitable for publication. Before a final decision can be made, however, the authors are requested to address several remaining issues through a minor revision:

1. Interpretation of gender differences: While the manuscript appropriately reports some observed differences in association patterns between girls and boys, several formal between-group comparisons were not statistically significant. Please further ensure throughout the Abstract, Results, Discussion, and Conclusions that interpretations remain cautious and do not overstate gender-specific effects where statistical evidence for group differences is limited.

2. Data availability statement: Please revise the Data Availability Statement to ensure full compliance with PLOS ONE data-sharing requirements. In particular, the justification for restrictions on data access should be clarified further, including a more explicit explanation of the ethical or legal constraints preventing public deposition of the dataset and the exact conditions under which data access may be granted.

3. Classroom climate measure: The use of a single-item measure for classroom climate is acknowledged as a limitation. Please strengthen the methodological justification for this choice in the Methods section and briefly elaborate on the implications this may have for interpretation of the findings.

4. Relevance of older data collection period:As the data were collected in 2013, please further clarify in the Discussion why the findings remain relevant to contemporary school contexts and current educational or physical activity practices.

5. Please carefully proofread the manuscript once more for clarity, consistency of terminology, and minor grammatical or stylistic issues.

==============================

Please submit your revised manuscript by Jul 02 2026 11:59PM. If you will need more time than this to complete your revisions, please reply to this message or contact the journal office at plosone@plos.org. When you're ready to submit your revision, log on to https://www.editorialmanager.com/pone/ and select the 'Submissions Needing Revision' folder to locate your manuscript file.

Please include the following items when submitting your revised manuscript:

- A letter that responds to each point raised by the academic editor and reviewer(s). You should upload this letter as a separate file labeled 'Response to Reviewers'.
- A marked-up copy of your manuscript that highlights changes made to the original version. You should upload this as a separate file labeled 'Revised Manuscript with Track Changes'.
- An unmarked version of your revised paper without tracked changes. You should upload this as a separate file labeled 'Manuscript'.

If you would like to make changes to your financial disclosure, please include your updated statement in your cover letter. Guidelines for resubmitting your figure files are available below the reviewer comments at the end of this letter.

If applicable, we recommend that you deposit your laboratory protocols in protocols.io to enhance the reproducibility of your results. Protocols.io assigns your protocol its own identifier (DOI) so that it can be cited independently in the future. For instructions see: https://journals.plos.org/plosone/s/submission-guidelines#loc-laboratory-protocols. Additionally, PLOS ONE offers an option for publishing peer-reviewed Lab Protocol articles, which describe protocols hosted on protocols.io. Read more information on sharing protocols at https://plos.org/protocols?utm_medium=editorial-email&utm_source=authorletters&utm_campaign=protocols.

As the corresponding author, your ORCID iD is verified in the submission system and will appear in the published article. PLOS supports the use of ORCID, and we encourage all coauthors to register for an ORCID iD and use it as well. Please encourage your coauthors to verify their ORCID iD within the submission system before final acceptance, as unverified ORCID iDs will not appear in the published article. *Only*  the individual author can complete the verification step; PLOS staff *cannot*  verify ORCID iDs on behalf of authors.

We look forward to receiving your revised manuscript.

Kind regards,

Georgian Badicu, Ph.D

Academic Editor

PLOS One

Journal Requirements:

If the reviewer comments include a recommendation to cite specific previously published works, please review and evaluate these publications to determine whether they are relevant and should be cited. There is no requirement to cite these works unless the editor has indicated otherwise.

Please review your reference list to ensure that it is complete and correct. If you have cited papers that have been retracted, please include the rationale for doing so in the manuscript text, or remove these references and replace them with relevant current references. Any changes to the reference list should be mentioned in the rebuttal letter that accompanies your revised manuscript. If you need to cite a retracted article, indicate the article’s retracted status in the References list and also include a citation and full reference for the retraction notice.

Additional Editor Comments:

The revised manuscript has improved substantially and addresses the majority of the reviewers’ and editor’s previous concerns. The study addresses an important and timely topic, and the analyses are generally appropriate and carefully conducted. The manuscript is now close to being suitable for publication.

Before a final decision can be made, however, the authors are requested to address several remaining issues through a minor revision:

1. Interpretation of gender differences: While the manuscript appropriately reports some observed differences in association patterns between girls and boys, several formal between-group comparisons were not statistically significant. Please further ensure throughout the Abstract, Results, Discussion, and Conclusions that interpretations remain cautious and do not overstate gender-specific effects where statistical evidence for group differences is limited.

2. Data availability statement: Please revise the Data Availability Statement to ensure full compliance with PLOS ONE data-sharing requirements. In particular, the justification for restrictions on data access should be clarified further, including a more explicit explanation of the ethical or legal constraints preventing public deposition of the dataset and the exact conditions under which data access may be granted.

3. Classroom climate measure: The use of a single-item measure for classroom climate is acknowledged as a limitation. Please strengthen the methodological justification for this choice in the Methods section and briefly elaborate on the implications this may have for interpretation of the findings.

4. Relevance of older data collection period:As the data were collected in 2013, please further clarify in the Discussion why the findings remain relevant to contemporary school contexts and current educational or physical activity practices.

5. Please carefully proofread the manuscript once more for clarity, consistency of terminology, and minor grammatical or stylistic issues.

[Note: HTML markup is below. Please do not edit.]

Reviewers' comments:

Reviewer's Responses to Questions

**Comments to the Author**

1. If the authors have adequately addressed your comments raised in a previous round of review and you feel that this manuscript is now acceptable for publication, you may indicate that here to bypass the “Comments to the Author” section, enter your conflict of interest statement in the “Confidential to Editor” section, and submit your "Accept" recommendation.

Reviewer #4: All comments have been addressed

**********

2. Is the manuscript technically sound, and do the data support the conclusions?

The manuscript must describe a technically sound piece of scientific research with data that supports the conclusions. Experiments must have been conducted rigorously, with appropriate controls, replication, and sample sizes. The conclusions must be drawn appropriately based on the data presented.

Reviewer #4: Yes

**********

3. Has the statistical analysis been performed appropriately and rigorously?

Reviewer #4: Yes

**********

4. Have the authors made all data underlying the findings in their manuscript fully available?

The PLOS Data policy requires authors to make all data underlying the findings described in their manuscript fully available without restriction, with rare exception (please refer to the Data Availability Statement in the manuscript PDF file). The data should be provided as part of the manuscript or its supporting information, or deposited to a public repository. For example, in addition to summary statistics, the data points behind means, medians and variance measures should be available. If there are restrictions on publicly sharing data—e.g. participant privacy or use of data from a third party—those must be specified.

Reviewer #4: Yes

**********

5. Is the manuscript presented in an intelligible fashion and written in standard English?

PLOS ONE does not copyedit accepted manuscripts, so the language in submitted articles must be clear, correct, and unambiguous. Any typographical or grammatical errors should be corrected at revision, so please note any specific errors here.

Reviewer #4: Yes

**********

6. Review Comments to the Author

Please use the space provided to explain your answers to the questions above. You may also include additional comments for the author, including concerns about dual publication, research ethics, or publication ethics. (Please upload your review as an attachment if it exceeds 20,000 characters)

Reviewer #4: The Reviewer thanks the Authors for responding thoughtfully to the prior comment. A couple of things to address briefly:

1. Was the classroom climate item reverse-coded? Please clarify

2. The Reviewer believes that altering the title to "Gendered patterns in the associations..." is more fitting and would match the conclusion by the Authors better (i.e., patterns were different, but not always statistically different between gender)

3. For Table 4, just make sure the direction of Cohen's d are all correct

4. For Tables 5 and 6, upper should probably be 97.5% rather than 2.5%. Conventionally, the Reviewer recommends 95% CI lower and upper.

5. From the Reviewer's scientific education and bias, a p-value should never be 0.000 - please report at p < 0.001

**********

7. PLOS authors have the option to publish the peer review history of their article (what does this mean?). If published, this will include your full peer review and any attached files.

If you choose “no”, your identity will remain anonymous but your review may still be made public.

**Do you want your identity to be public for this peer review?**  For information about this choice, including consent withdrawal, please see our Privacy Policy.

Reviewer #4: **Yes:**  Shane J.T. Balthazaar

**********

[NOTE: If reviewer comments were submitted as an attachment file, they will be attached to this email and accessible via the submission site. Please log into your account, locate the manuscript record, and check for the action link "View Attachments". If this link does not appear, there are no attachment files.]

To ensure your figures meet our technical requirements, please review our figure guidelines: https://journals.plos.org/plosone/s/figures

You may also use PLOS’s free figure tool, NAAS, to help you prepare publication quality figures: https://journals.plos.org/plosone/s/figures#loc-tools-for-figure-preparation.

NAAS will assess whether your figures meet our technical requirements by comparing each figure against our figure specifications.

# Author comment: Author response to Decision Letter 3

**DOI:** 10.1371/journal.pone.0351971.r006

29 May 2026

We sincerly thank the reviewer for the careful evaluation of our manuscript and for the constructive comments provided. We have carefully considered all comments and have revised the manuscript accordingly. A detailed, point-by-point response is provided in a separate document, and all corresponding changes have been indicated in the revised manuscript.

Attachment Submitted filename: Response_to_Reviewers_auresp_3.pdf

# Editor report: Decision Letter 3

**DOI:** 10.1371/journal.pone.0351971.r007

**Authors:** Georgian Badicu, Georgian Badicu, Georgian Badicu, Georgian Badicu

3 Jun 2026

Gendered patterns in the associations between in-school physical activity, classroom peer ecology, and academic achievement among primary school students

PONE-D-25-55757R3

Dear Dr. Byman,

We’re pleased to inform you that your manuscript has been judged scientifically suitable for publication and will be formally accepted for publication once it meets all outstanding technical requirements.

Within one week, you’ll receive an e-mail detailing the required amendments. When these have been addressed, you’ll receive a formal acceptance letter and your manuscript will be scheduled for publication.

An invoice will be generated when your article is formally accepted. Please note, if your institution has a publishing partnership with PLOS and your article meets the relevant criteria, all or part of your publication costs will be covered. Please make sure your user information is up-to-date by logging into Editorial Manager at Editorial Manager® and clicking the ‘Update My Information' link at the top of the page. For questions related to billing, please contact billing support.

If your institution or institutions have a press office, please notify them about your upcoming paper to help maximize its impact. If they’ll be preparing press materials, please inform our press team as soon as possible -- no later than 48 hours after receiving the formal acceptance. Your manuscript will remain under strict press embargo until 2 pm Eastern Time on the date of publication. For more information, please contact onepress@plos.org.

Kind regards,

Georgian Badicu, Ph.D

Academic Editor

PLOS One

Additional Editor Comments (optional):

Reviewers' comments:

# Editor report: Acceptance letter

**DOI:** 10.1371/journal.pone.0351971.r008

**Authors:** Georgian Badicu, Georgian Badicu, Georgian Badicu, Georgian Badicu

PONE-D-25-55757R3

PLOS One

Dear Dr. Byman,

I'm pleased to inform you that your manuscript has been deemed suitable for publication in PLOS One. Congratulations! Your manuscript is now being handed over to our production team.

At this stage, our production department will prepare your paper for publication. This includes ensuring the following:

* All references, tables, and figures are properly cited

* All relevant supporting information is included in the manuscript submission,

* There are no issues that prevent the paper from being properly typeset

You will receive further instructions from the production team, including instructions on how to review your proof when it is ready. Please keep in mind that we are working through a large volume of accepted articles, so please give us a few days to review your paper and let you know the next and final steps.

Lastly, if your institution or institutions have a press office, please let them know about your upcoming paper now to help maximize its impact. If they'll be preparing press materials, please inform our press team within the next 48 hours. Your manuscript will remain under strict press embargo until 2 pm Eastern Time on the date of publication. For more information, please contact onepress@plos.org.

You will receive an invoice from PLOS for your publication fee after your manuscript has reached the completed accept phase. If you receive an email requesting payment before acceptance or for any other service, this may be a phishing scheme. Learn how to identify phishing emails and protect your accounts at https://explore.plos.org/phishing.

If we can help with anything else, please email us at customercare@plos.org.

Thank you for submitting your work to PLOS ONE and supporting open access.

Kind regards,

PLOS ONE Editorial Office Staff

on behalf of

Dr. Georgian Badicu

Academic Editor

PLOS One
